# Borate-Bridged Protolipids: A Prebiotic Route to Abiotic Membranes †

**DOI:** 10.3390/life16050714

**Published:** 2026-04-22

**Authors:** Valery M. Dembitsky, Alexander O. Terent’ev, Ion Romulus I. Scorei

**Affiliations:** 1 Bio-Pharm Laboratories, 23615 El Toro Rd., Lake Forest, CA 92630, USA; 2 N.D. Zelinsky Institute of Organic Chemistry, Russian Academy of Sciences, 47 Leninsky Prospect, Moscow 119334, Russia; terentev@ioc.ac.ru; 3 Department of Biochemistry, BioBoron Research Institute, S.C. Natural Research S.R.L., 31B Dunării Street, 207465 Podari, Romania; romulus_ion@yahoo.com

**Keywords:** abiotic, membrane, borates, ribose, carbohydrates, conjugates, boronolipids

## Abstract

The emergence of membrane boundaries represents a decisive transition in the origin of life, yet the molecular nature of the earliest abiotic membranes remains uncertain. Existing models based on simple fatty acids, while experimentally tractable, often lack the environmental robustness required under fluctuating prebiotic conditions. Furthermore, the absence of clear pathways linking primitive amphiphiles to later phospholipid systems highlights the need for chemically continuous intermediate frameworks. Here, we explore borate-bridged amphiphile–carbohydrate conjugates as plausible intermediates between simple prebiotic surfactants and modern lipid bilayers. These conjugates arise from low-molecular-weight polyols—including glycerol, butane-1,2,3,4-tetraol, pentane-1,2,3,4,5-pentaol, and hexane-1,2,3,4,5,6-hexitol—reacting with long-chain alkyl ethers and borate species under alkaline conditions, enabling reversible coupling to ribose and other vicinal diol-containing sugars. This chemistry integrates three essential properties for early compartmentalization: hydrolytically robust ether-linked hydrophobic domains, multivalent and highly hydrated headgroups, and environmentally responsive borate coordination. Comparative physicochemical analysis suggests that single-tail alkylglycerol derivatives preferentially form micelles and interfacial films, while di- and tri-tail tetritol and pentitol conjugates favor lamellar assemblies and vesicle formation across realistic prebiotic pH and salinity ranges. Hexitol-based systems, particularly those bearing three hydrophobic chains, may act as membrane-stabilizing components that enhance rigidity and reduce permeability under extreme conditions. We propose that heterogeneous mixtures dominated by two-tail polyol diethers, supplemented by tri-tail stabilizers and surface-active alkylglycerols, could provide mechanically robust, pH-tunable, and sugar-decorated abiotic membranes. Such borate-mediated amphiphiles offer a chemically coherent framework linking carbohydrate stabilization, ether lipid persistence, and dynamic self-assembly, potentially representing a transitional stage in the evolutionary pathway from primitive amphiphilic films to biologically encoded membranes.

## 1. Introduction

The origin of biological membranes remains a central problem in prebiotic chemistry [1,2,3,4,5,6,7,8,9,10,11]. Compartmentalization is essential for metabolism and evolution, yet the identity of early membrane-forming amphiphiles is unresolved [12,13,14,15,16,17]. While fatty acids are widely proposed as prebiotic surfactants, they are fragile under saline conditions and sensitive to divalent cations and temperature. These limitations suggest that more robust amphiphiles with improved interfacial organization may have been critical in forming persistent protomembranes [1,8,9,18,19,20].

Ether-linked amphiphiles derived from low–molecular-weight polyols are promising candidates. Polyols such as ethylene glycol, glycerol, and higher sugar alcohols can arise from abiotic carbonyl chemistry and provide multiple hydroxyl groups for reactivity. Partial alkylation yields amphiphiles resembling modern ether lipids, with enhanced stability under alkaline, thermal, and mineral-rich conditions. These properties position polyol-derived ether lipids as plausible intermediates between simple surfactants and biological membranes [1,2,21,22,23,24,25,26,27,28].

Boron introduces an additional geochemical dimension. Borate species form cyclic esters with vicinal diols, stabilizing carbohydrates such as ribose, which is otherwise unstable under prebiotic conditions. Through selective complexation, borate can bias ribose away from degradation pathways [29,30,31,32,33,34,35]. Beyond solution chemistry, borate interactions may enable reversible ribose capture at interfaces, allowing membrane-like assemblies to concentrate sugars prior to enzymatic processes. Under alkaline conditions, borate–diol interactions form tetrahedral bis(diolato) complexes, including spirocyclic structures [36,37,38,39,40,41].

Applied to amphiphilic polyols, this chemistry yields borate-bridged amphiphile–carbohydrate conjugates, where hydrophobic anchors are linked to hydrated sugar headgroups via boron-mediated bridges. These systems combine key features for early membranes: stable ether-linked tails, hydrogen-bonding polyol headgroups, and borate-mediated responsiveness. Unlike fatty acids, they are expected to form persistent micelles, films, or bilayers depending on structure, while enabling carbohydrate recruitment without enzymatic glycosylation [1,2,3,4,5,8,19,25].

Their behavior depends on polyol structure: tetritols favor bilayers with tunable curvature, while higher polyols enhance crosslinking and stability. Borate complexation is pH- and ionic-strength dependent, allowing dynamic responses to environmental gradients such as hydrothermal or evaporative settings. Thus, these systems integrate membrane formation, sugar stabilization, and environmental responsiveness within a single framework [1,2,25,33,34].

In this review, we examine the chemistry, self-assembly, and geochemical plausibility of borate-bridged amphiphile–carbohydrate systems as components of abiotic membranes. By linking ether lipid chemistry with borate–diol interactions, we propose a model in which early membranes functioned as chemically active, responsive interfaces, bridging simple amphiphile aggregates and modern lipid bilayers.

## 2. Alkyl Glycerol Ethers in Nature and Evolution

Batyl, selachyl, and chimyl alcohols are naturally occurring alkyl glycerol ethers widely distributed in marine organisms, freshwater systems, and sedimentary deposits. These single-tail ether lipids represent structurally simple yet chemically robust amphiphiles that occupy a unique intersection between biochemistry, medicine, and prebiotic chemistry [2,21,22,23,24,25,26,27]. Their biological activities—particularly immunomodulatory and anticancer properties—have drawn considerable attention in medical research [42,43]. At the same time, their chemical stability, ability to form amphiphilic assemblies, and capacity to form borate complexes through vicinal diol chemistry position them as compelling candidates in discussions of membrane evolution and abiotic compartment formation. This review examines their chemical structure, natural occurrence, biological activity, physicochemical behavior, boron complexation, and potential evolutionary implications.

### 2.1. Chemical Structure and Classification

Batyl alcohol (1-O-octadecyl-glycerol), selachyl alcohol (1-O-octadecenyl-glycerol), and chimyl alcohol (1-O-hexadecyl-glycerol) are alkyl glycerol ethers, also referred to as alkylglycerols (AKGs). They consist of: (i) A glycerol backbone; (ii) A single long alkyl chain attached via an ether bond at the *sn*-1 position, (iii) Two remaining hydroxyl groups at *sn*-2 and *sn*-3 [44,45,46,47,48,49].

Structurally, they are *lyso*-ether lipids—analogous to lysophospholipids but containing an ether linkage instead of an ester bond. The ether bond confers increased hydrolytic stability compared to ester-linked lipids. Selachyl alcohol contains an unsaturation (C18:1), which introduces fluidity into assemblies; batyl and chimyl are saturated (C18:0 and C16:0, respectively) [2,44,45,46,47,48,49]. The presence of two free hydroxyl groups makes these molecules not only amphiphilic but also chemically reactive toward diol-binding agents such as borate.

### 2.2. Natural Occurrence

Alkyl glycerol ethers are widely distributed in marine organisms, particularly: (i) Sharks and elasmobranch fish (notably shark liver oil) [24,25,50,51]; (ii) Marine invertebrates, certain sponges, and microorganisms [42,43]. They have also been detected in: (i) Marine and freshwater sediments [52,53]; (ii) Fossil lipid deposits [42], and (iii) Petroleum-associated organic matter [42,54].

Their persistence in sediments reflects the relative chemical stability of ether bonds compared to esters. This stability has implications not only for geochemistry but also for evolutionary membrane chemistry.

### 2.3. Biological and Medical Significance

These alkyl glycerol ethers are biologically active compounds with documented effects, including: (i) immunomodulatory activity; (ii) enhancement of hematopoiesis; (iii) antitumor and anticancer effects; (iv) anti-inflammatory properties; and (v) antimicrobial activity [42,43,47].

One reason for their biomedical interest is their role as precursors in ether lipid metabolism, including plasmalogen biosynthesis. Plasmalogens are vinyl ether phospholipids implicated in membrane structure, oxidative stress response, and signaling pathways [55,56,57,58].

In oncology research, ether lipids have attracted attention because: (i) tumor cell membranes often exhibit altered ether lipid composition; (ii) ether lipids can influence membrane microdomain organization; and (iii) modulation of ether lipid metabolism affects tumor progression in certain models. Thus, batyl, selachyl, and chimyl alcohols represent both structural lipids and functional modulators of cellular physiology [42,43,55,56,57,58].

### 2.4. Physicochemical Properties and Self-Assembly

As single-tail amphiphiles, these compounds exhibit properties characteristic of lysolipids: (i) Formation of micelles above critical aggregation concentrations; (i) Strong surface activity; (iii) Ability to form monolayers at interfaces, (iv) Potential to stabilize emulsions and surface films [22,23,24,25,42,43].

Because they possess only one hydrophobic chain, they generally favor high-curvature aggregates rather than stable bilayers when alone. However, in mixtures with fatty acids or diacyl lipids, they can: (i) Modify curvature; (ii) Stabilize or destabilize membranes, and (iii) Influence permeability and elasticity. Selachyl alcohol, due to its unsaturation, increases membrane fluidity and decreases packing density relative to saturated batyl and chimyl alcohols [44,45,46,47].

### 2.5. Borate Complex Formation

A particularly underexplored aspect of these molecules is their capacity to form borate diol complexes. The two adjacent hydroxyl groups on the glycerol backbone constitute a vicinal diol system capable of interacting with boric acid or borate anions under alkaline conditions [59,60,61,62,63].

In such environments: (i) Boron forms cyclic borate esters with the diol moiety; (ii) Tetrahedral bis(diolato)borate complexes may form, and (iii) Spiroborate structures are possible if additional diol-containing molecules (e.g., sugars) participate [1,20].

**Figure 1:**
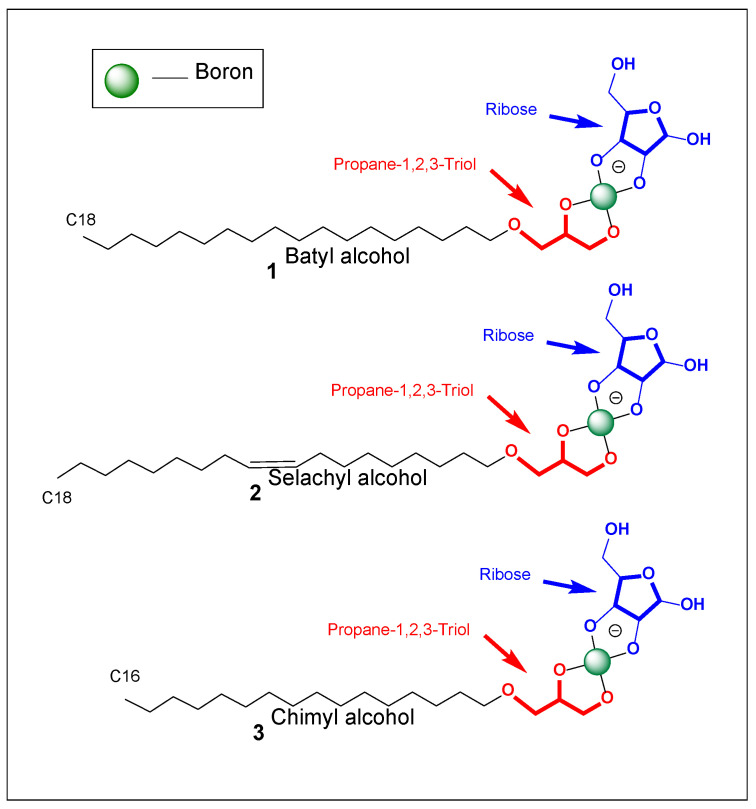
Bioactive alkyl glycerides: batyl, selachyl, and chimyl alcohols, and their borate-bridged amphiphile–carbohydrate conjugates. These naturally occurring alkyl glycerol ethers consist of a glycerol backbone bearing a single long-chain alkyl ether at the sn-1 position and two free hydroxyl groups at sn-2 and sn-3, conferring both amphiphilic character and diol reactivity. The structural differences among them—chain length and degree of unsaturation—strongly influence their membrane behavior, biological activity, and capacity to form borate-mediated complexes with carbohydrates.

When ribose or other carbohydrates are present, borate can bridge between the glycerol diol and the sugar diol, generating borate-bridged amphiphile–carbohydrate conjugates (see Figure 1). These complexes create enlarged, structured, and highly hydrated headgroups while retaining the hydrophobic tail. This chemistry introduces: (i) pH-dependent amphiphile behavior; (ii) Dynamic sugar recruitment to interfaces, and (iii) Potential crosslinking under certain conditions. Such systems may have relevance in prebiotic chemistry [30,31,64,65,66].

### 2.6. Evolutionary and Prebiotic Implications

Ether lipids are a defining feature of archaeal membranes and are associated with resilience to extremes of temperature, pH, and salinity [1,2]. The occurrence of natural alkyl glycerol ethers (e.g., batyl and chimyl alcohols, Figure 1) shows that simple ether-linked amphiphiles are both chemically feasible and biologically compatible. This supports the idea that ether-linked scaffolds could have acted as robust intermediates in membrane evolution, potentially preceding fully developed phospholipid biosynthesis.

From a prebiotic perspective, alkyl glycerol ethers and related polyol-derived amphiphiles address key limitations of fatty-acid vesicle models [1]: (i) greater hydrolytic stability than esters; (ii) strong tendencies to form films, micelles, and, when double-tailed, bilayer-like structures; (iii) vicinal diol motifs enabling borate coordination; and (iv) compatibility with alkaline, mineral-rich environments. These advantages extend to higher polyols (e.g., tetritols), which retain diol functionality while supporting more robust two-tailed architectures.

An important implication is that early chemical selection may have favored structural performance over abundance. Even if less prevalent, polyols capable of forming stable, low-permeability assemblies could have been preferentially retained [1]. In this view, membrane persistence acts as a selective filter, favoring amphiphiles that remain stable across variable pH, ionic strength, and temperature.

Within this framework, boron provides a mechanism for chemical responsiveness and interfacial organization. In borate-rich environments, diol- or tetraol-containing amphiphiles could reversibly bind carbohydrates, forming borate-bridged protolipids with hydrated headgroups. This offers a route for carbohydrate recruitment (e.g., ribose-like molecules) without enzymatic glycosylation, while maintaining environmental sensitivity through borate speciation. Such systems are best described by a window/threshold model, where stable assembly occurs only within specific physicochemical conditions.

Overall, borate-bridged ether amphiphiles represent plausible intermediates between fatty-acid assemblies and modern phospholipid membranes. They provide a chemically coherent framework linking robustness, reversible coordination chemistry, and interfacial molecular organization in both prebiotic and biological contexts.

### 2.7. Prospects of Alkyl Glycerol Ethers

Batyl (**1**), selachyl (**2**), and chimyl (**3**) alcohols occupy a unique position at the interface of natural product chemistry, medicine, and evolutionary biochemistry. Structurally simple yet versatile, these alkyl glycerol ethers combine amphiphilic self-assembly, ether stability, and diol reactivity [1,2]. Their biological roles highlight compatibility with living systems, while their chemistry suggests relevance to abiotic membrane formation via borate–diol interactions (Figure 1, and Table 1 and Table 2).

These naturally occurring alkyl glycerol ethers feature a glycerol backbone with a single long hydrophobic chain linked by a stable ether bond and two vicinal hydroxyl groups [24,42,43,44,45]. Under alkaline conditions, these hydroxyls react with boric acid or borate to form cyclic borate esters. In the presence of ribose or other 1,2-diols, boron can bridge the amphiphile and sugar, yielding borate-bridged amphiphile–carbohydrate conjugates, typically as tetrahedral bis(diolato)borate or spiroborate structures. This coordination expands and organizes the headgroup, increases hydration and hydrogen bonding, and may introduce pH-dependent charge. These dynamic covalent amphiphiles depend on borate speciation, ionic strength, and pH for stability [67,68]. The ether linkage remains hydrolytically robust, preserving the hydrophobic anchor, while the borate bridge enables reversible, environmentally responsive coupling to carbohydrates.

Physicochemically (Table 1), these conjugates behave as single-tail amphiphiles with enlarged, hydrated headgroups that favor micelle formation and strong interfacial activity. Increased headgroup area promotes cone-shaped geometry, supporting micelles above a critical aggregation concentration and dense interfacial monolayers. In mixtures with fatty acids or diacyl lipids, they modulate curvature, permeability, and membrane mechanics, acting as surface-active components that recruit carbohydrates. The pH-responsive borate linkage allows reversible sugar binding and lateral headgroup interactions, influencing film rigidity and aggregate morphology. Overall, these borate-bridged systems represent robust, tunable amphiphiles capable of forming micelles and interfacial assemblies, providing a plausible bridge between simple ether lipids and carbohydrate-containing membrane systems in biological and prebiotic contexts.

### 2.8. Thermodynamic Analysis of Micelle Formation and pH-Dependent Binding Constants

From a thermodynamic standpoint, micellization of batyl/selachyl/chimyl-derived borate–sugar conjugates can be treated with the same framework used for surfactants, but with two important twists: (i) the headgroup is much more hydrated and often partially charged, and (ii) the headgroup chemistry is coupled to an equilibrium (borate–diol–sugar binding (Table 1). For a single-tailed amphiphile, micelle formation occurs above the critical micelle concentration (CMC), and the standard free energy of micellization per mole of monomer can be approximated by ΔG°mic ≈ RT ln(X_CMC), where X_CMC is the CMC expressed as a mole fraction (for dilute aqueous solutions, X_CMC ≈ CMC/55.5).

More negative ΔG°_mic means a stronger driving force and lower CMC. The hydrophobic tail strongly favors micellization (hydrophobic effect), whereas a bulky/charged headgroup raises the CMC by increasing steric/electrostatic penalties in the aggregate. In system, borate–sugar complexation typically increases headgroup area and hydration, which tends to raise the CMC and favors higher-curvature aggregates (smaller micelles) rather than bilayers. However, the same complexation can also increase colloidal stability once micelles exist, because strongly hydrated headgroups reduce aggregation between micelles and suppress precipitation. Ionic strength becomes a key thermodynamic control: screening of headgroup charge reduces electrostatic repulsion and can lower the CMC (making ΔG°_mic more negative), while low ionic strength can keep the CMC higher and favor more dispersed states. Selachyl-derived conjugates (with one double bond) generally have a slightly weaker hydrophobic packing term than saturated batyl/chimyl analogs, often translating into more fluid, dynamic micelles and sometimes a modest shift in CMC upward or downward depending on how chain disorder competes with hydration (experimentally, this is typically resolved via surface tension or isothermal titration calorimetry).

For the pH-dependent formation of the borate-bridged amphiphile–carbohydrate conjugate, a useful minimal model is a coupled equilibrium between boron speciation and diol binding. First, boric acid/borate speciation can be written as B(OH)_3_ + OH^−^ ⇌ B(OH)_4_^−^, with an apparent pK_a_ ≈ 9–9.3 in dilute water (and shifted by ionic strength). The fraction of borate available is f_B_ = 1/(1 + 10^(pK_a_ − pH)). Second, borate binding to a diol pair can be summarized as B* + L ⇌ B–L, where L is the vicinal diol ligand (glycerol-diol, sugar-diol), and the equilibrium constant depends strongly on diol geometry. In the simplest “bridging” picture, the amphiphile diol (A) first forms a borate ester (A–B), then the sugar diol (S) binds to yield a ternary complex (A–B–S). If we denote the intrinsic stepwise constants as K_1_ for A binding and K_2_ for S binding to the preformed A–B complex, then the *conditional* observed stability at a given pH scales approximately as K_obs_(pH) ≈ f_B_ · K_1_ · K_2_ (more detailed treatments add protonation states of the diols and competition by water/other polyols, but this captures the dominant pH lever: complexation rises steeply as f_B_ increases near and above the pK_a_). Thermodynamically, complex formation contributes ΔG°_bind = −RT ln K_obs_(pH), so every unit increase in log_10_K_obs lowers ΔG°_bind by ≈ 5.7 kJ/mol at 298 K. Because the conjugate fraction controls headgroup size/charge, we effectively get a “chemical feedback” into assembly: as pH rises, K_obs increases → more conjugate → larger/possibly more charged headgroups → higher curvature and often higher CMC, unless ionic strength compensates by screening repulsion. In practice, this predicts a tunable phase behavior: near-neutral pH yields weaker conjugation and more compact headgroups (micelles still possible, but less sugar-decorated), while mildly to strongly alkaline conditions shift the equilibrium toward sugar-decorated borate complexes, producing more hydrated, more responsive micelles and surface films whose stability and size should be strongly salt-dependent.

Borate-bridged amphiphile–carbohydrate conjugates derived from batyl, selachyl, and chimyl alcohols represent a chemically and physically versatile class of compounds (Figure 1). The combination of ether-linked hydrophobic stability, diol-mediated borate coordination, and carbohydrate incorporation creates amphiphiles that are: (i) Structurally robust; (ii) Environmentally responsive; (iii) Capable of micelle and surface film formation, and (iv) Potentially relevant to prebiotic membrane evolution. These systems bridge modern biological lipid chemistry and plausible abiotic amphiphile behavior, offering a unified framework for understanding how simple ether lipids could evolve into chemically functional membrane components.

Batyl and selachyl alcohols are very naturally framed as “lyso-ether lipids” in the physicochemical sense: they carry one long hydrophobic chain (C18 in batyl; C18:1 in selachyl) attached by an ether bond to a glycerol backbone, while retaining polar hydroxyl functionality that can be further “functionalized” by borate binding and sugar (ribose) conjugation [69,70,71,72]. That combination makes them classic single-tailed amphiphiles, and in soft-matter terms single-tailed amphiphiles are exactly the class of molecules that most readily form micelles and surface-active films. Unlike double-tailed lipids, which strongly favor low-curvature bilayers, a single tail plus a comparatively large and hydrated headgroup gives a cone-shaped geometry; cone-shaped amphiphiles pack efficiently into high-curvature aggregates (spherical or ellipsoidal micelles) and into monolayers at interfaces.

The “lysolipid analogy” is more than a metaphor because it predicts behavior. In modern cells, lysophospholipids (one tail removed from a phospholipid, Figure 1) have a high spontaneous curvature and are powerful surfactants that destabilize bilayers and promote micelles or pores. Batyl/selachyl alcohols, especially when their headgroups are expanded by borate and ribose, behave similarly: the effective headgroup area increases due to multiple hydroxyls, borate coordination, and sugar hydration, while the hydrophobic volume remains that of a single chain [30,58,62,64]. This pushes the packing parameter downward, favoring micelles and monolayers rather than stable bilayers. In practice, this means batyl/selachyl systems are expected to form stable micellar solutions above a critical aggregation concentration, and to adsorb strongly to interfaces such as air–water, oil–water, or mineral–water boundaries, generating surface films that can resemble “membrane-like” barriers even though they are not bilayers.

Borate and ribose specifically make these assemblies more “membrane-like” in function because they transform the headgroup from merely hydrophilic into one that is structured, chemically interactive, and condition-dependent. Borate binds vicinal diols on both the glycerol moiety and ribose, forming a cyclic complex that is typically favored under alkaline conditions. When ribose is incorporated into the headgroup through borate coordination, the polar region becomes larger, more hydrogen-bond-rich, and often more charged or strongly polarized (depending on borate speciation and counterions). This has two consequences: first, it tends to stabilize micelles (because the headgroups strongly prefer to remain solvated and repel coalescence); and second, it can create a highly hydrated interfacial layer that behaves like a primitive “glycocalyx-like” surface—capable of binding ions, organizing water, and selectively interacting with other diols or polyols in solution [29,30,58,62,64].

Surface films are particularly important for prebiotic and “abiotic membrane” considerations because they do not require a bilayer to exhibit compartment-like behavior. A dense monolayer of single-tailed amphiphiles can reduce permeability to certain solutes, stabilize droplets and bubbles, coat mineral pores, and form interfacial boundaries that concentrate organics at the surface. Batyl and selachyl alcohols are strong candidates for this role because their long alkyl chains anchor them at interfaces, while the glycerol/borate/sugar headgroup remains strongly hydrated and capable of forming lateral hydrogen-bonding networks. Even if such films are only one molecule thick, they can create persistent boundaries in environments rich in interfaces—foams, aerosols, emulsions, coacervate droplets, or pore-water surfaces—precisely the types of settings considered chemically productive in many origin-of-life models [73,74,75].

The difference between batyl and selachyl alcohols is also significant. Selachyl contains an unsaturation (C18:1), which typically increases chain disorder and lowers melting/packing temperature. This tends to make selachyl-derived micelles and films more fluid and dynamic at a given temperature than those derived from batyl. Batyl (saturated C18) tends to pack more tightly and can form more rigid assemblies, potentially resulting in lower permeability but also reduced adaptability. In mixed systems, a combination of saturated and unsaturated single-tailed ether lipids often produces films with tunable elasticity—an important property for maintaining stable but non-brittle interfaces.

Finally, the most important conceptual point is that single-tailed ether lipids with borate–ribose headgroups can act as feedstock and modifiers for more complex membrane evolution. On their own they favor micelles and monolayers; when mixed with fatty acids or double-tailed amphiphiles, they can (i) stabilize aggregates, (ii) modify curvature, and (iii) decorate surfaces with carbohydrate functionality through reversible borate binding. That makes them plausible early “surface-active organizers” that could have preceded robust bilayer membranes. They don’t need to be the final membrane architecture to be evolutionarily central: in an abiotic environment, molecules that form persistent micelles and films can create the first stable boundaries, the first concentration platforms, and the first chemically selective interfaces—setting the stage for later emergence of two-tailed lipids that yield true bilayer membranes [29,30,62,64].

## 3. Butane-1,2,3,4-Tetraol Scaffold and Their Properties

Can get surprisingly far with “math-first” physicochemical estimates for 3,4-bis(octadecyloxy)butane-1,2-diol–borate–ribose, even before doing experiments, as long as we’re explicit about assumptions and treat the borate–diol linkage as an equilibrium that changes with pH and salt [1]. I’ll walk through (i) molar mass, (ii) pH-dependent stability/speciation, and (iii) propensity to form films/monolayers and bilayers, each as a detailed paragraph.

The molar mass is the easiest place to start because it depends mainly on composition, not on equilibrium. The lipid scaffold named, 3,4-bis(octadecyloxy)butane-1,2-diol, can be approximated as butane-1,2,3,4-tetraol in which the 3-OH and 4-OH are etherified with two octadecyl (C_18_H_37_) chains, leaving the 1,2-diol free for borate binding. That gives the diether-diol backbone formula C_40_H_82_O_4_, which corresponds to an estimated molecular weight of ~627.1 g/mol. Ribose is C_5_H_10_O_5_ with MW ~150.1 g/mol. For the borate-linked conjugate, a common alkaline-water representation is a borate diester-type complex in which boron coordinates to two diol motifs (one from the butane-1,2-diol and one from ribose), giving a tetrahedral boron center whose net charge is often **−**1 at high pH. Under that simplifying assumption, the conjugate’s empirical formula is reasonably approximated as C_45_H_88_BO_9_**^−^**, with an estimated MW of ~784.0 g/mol. The key uncertainty is that boron–diol complexes can exist in multiple hydrated/protonation states (e.g., retaining an –OH on boron or being neutral vs. anionic depending on pH and counterions), so the “real” mass in solution may differ by small increments corresponding to ±H/±OH/associated counterion, but ~784 g/mol is a solid baseline for the main organic framework.

The stability versus pH can be modeled as a coupled equilibrium: (1) boron speciation (boric acid ⇌ borate) and (2) borate binding to cis/vicinal diols (tetritol-like diol and sugar diol). In fresh water at 25 °C, boric acid has an apparent pK_a_ near ~9.2 (values around 9.14–9.24 are commonly reported), meaning that above pH ~9–10 the fraction of boron present as borate rises steeply. In seawat~8.6 in artificial seawater-like ionic backgrounds, the apparent pK_a_ can shift downward (one source notes at salinity ~35 g/L), implying that “borate-available” conditions may occur at slightly lower pH in marine settings. A simple quantitative handle is the Henderson–Hasselbalch fraction of borate:

*f*_(borate)_ = 1/(1 + 10^(pK_a_ − pH)).

Using pK_a ≈ 9.24 (fresh water): f ≈ 0.05 at pH 8.0, 0.36 at pH 9.0, 0.85 at pH 10.0, and 0.98 at pH 11.0; using pK_a ≈ 8.6 (seawater-like): f ≈ 0.20 at pH 8.0, 0.72 at pH 9.0, 0.96 at pH 10.0. That math captures the first-order reason conjugate becomes “more stable” in alkaline environments: more borate is present to form diol complexes. The second-order reason is that borate–diol ester stability is well known to be pH-dependent and solvent/ionic-strength dependent, with stronger complexation as conditions favor borate ester formation. Importantly for specific case, there is literature indicating that ribose–borate complexes can be comparatively stable (even relative to other aldopentoses) and that borate complexation has been discussed as a plausible stabilizing/selection factor for ribose in prebiotic contexts—exact constants depend on conditions and the boron source (solution vs. mineral). So the most defensible “math-based” stability statement is not a single lifetime, but a stability window: the sugar–borate–lipid headgroup association should be weak/short-lived below roughly pH 8–9 (fresh water), strengthened markedly near pH ~9–10, and maximized at pH ≥ 10, while in seawater-like ionic media that strengthening may begin closer to pH ~8.5–9.5 because borate becomes significant sooner.

A practical implication of this speciation dependence is that ribose decoration of borate-bridged amphiphiles should exhibit a defined “binding window” as a function of pH and ionic strength. Accordingly, the fraction of ribose-decorated amphiphile—and its interfacial signatures (e.g., zeta potential, headgroup hydration, and aggregate curvature/vesicle size)—should co-vary with pH in a manner consistent with ^11^B NMR speciation and with salt-dependent screening of headgroup repulsion. This co-variation offers a straightforward experimental test of borate-mediated carbohydrate association at membrane-like interfaces.

**Figure 2:**
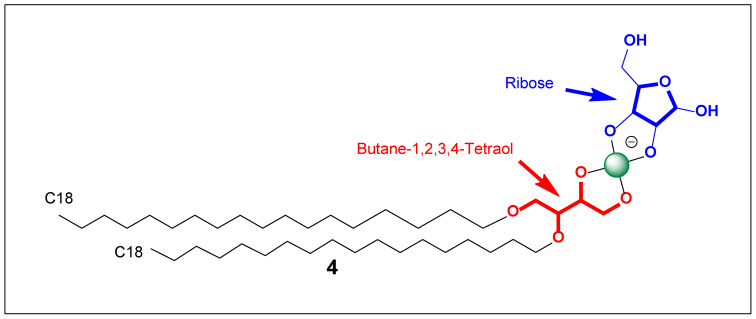
Structure of a borate-bridged amphiphile–carbohydrate conjugate based on the low-molecular-weight polyol, butane-1,2,3,4-tetraol. In this architecture, the tetritol backbone provides vicinal diol groups that coordinate with borate to form a cyclic bis(diolato)borate bridge linking the amphiphilic ether tail(s) to a carbohydrate moiety such as ribose. The borate center generates a structured and highly hydrated headgroup while preserving the hydrophobic domain required for self-assembly. This dual functionality enables the molecule to act as a pH-responsive amphiphile capable of forming micelles, surface films, or membrane-modifying assemblies under appropriate environmental conditions.

The ability to spontaneously form films (monolayers) and membranes can also be treated quantitatively using amphiphile packing and interfacial thermodynamics. The conjugate is effectively a double-tailed lipid (two C18 chains) with a very polar, hydrated headgroup (butane-1,2-diol + borate + ribose, Figure 2). Double-tailed amphiphiles are intrinsically biased toward bilayers and films because their hydrophobic volume is large and their shape tends toward cylindrical rather than conical. A standard “math tool” here is the critical packing parameter (CPP): CPP = v/(a_0_·*l*), where v is the hydrophobic tail volume, l is the tail length, and a_0_ is the effective headgroup area. For a C18 chain, Tanford-type estimates give l ≈ 24 Å and v ≈ 510 Å^3^ per tail; two tails give *v*_total ≈ 1020 Å^3^. If the effective headgroup area a_0_ is ~70 Å^2^ (more compact headgroup—lower pH, less borate/sugar loading), CPP ≈ 1020/(70·24) ≈ 0.61, which lies in the bilayer/vesicle-favoring regime (roughly CPP ~0.5–1). If a_0_ expands to ~100–120 Å^2^ (high pH, strong borate binding, high sugar occupancy, strong hydration/charge), CPP drops to ~0.35–0.43, pushing toward higher-curvature aggregates (smaller vesicles, wormlike micelles, or mixed phases) unless electrostatic repulsion is screened. This yields a clear, testable prediction: lower pH/lower sugar loading → larger, more stable lamellar films, whereas higher pH/higher sugar loading → greater curvature and smaller vesicles, with salt (ionic strength) shifting the system back toward bilayers by screening headgroup repulsion and effectively reducing a_0_ through diminished electrostatic expansion.

This same logic supports film formation at interfaces: amphiphiles with two long chains almost always exhibit strong surface activity and can form monolayers at air–water or solid–water interfaces. The borate–sugar headgroup (4, Figure 2) should further enhance adhesion to hydrophilic surfaces through hydrogen bonding and ion–dipole interactions, making abiotic “membrane-on-mineral” films plausible—especially where pH and ionic conditions stabilize borate conjugation. The key caveat is that, because the borate–sugar linkage is reversible, the surface film may persist while the sugar decoration exchanges, producing a membrane that is structurally stable but chemically adaptive.

If want the next step in the same quantitative spirit, the most informative “minimal experimental dataset” to parameterize a real stability model would be: (1) pH titration with and without sugar while monitoring boron speciation (often via ^11^B NMR) to extract conditional formation constants, (2) zeta potential vs. pH/salt to quantify headgroup charge, and (3) surface pressure–area isotherms (Langmuir trough) or QCM-D on silica/mica to directly measure spontaneous film formation and stability under changing pH/salinity. Those three measurements convert the qualitative chemistry into numbers we can actually plug back into equilibrium and packing models.

That structural name is a mouthful, but chemically it conveys a very clear picture: we are describing a double-tailed amphiphile in which a sugar-like furanose unit is “locked” to boron through cyclic boronate/borate rings, forming a spiro-borate architecture (a rigid, multidentate boron–diol framework), while the hydrophobic domain is provided by two C18 ether chains. In prebiotic (abiotic) terms, this combination is powerful because it couples (1) a strong, low-entropy driving force for self-assembly (two long tails) with (2) a headgroup that is both highly polar and structurally preorganized (boron-bridged diol rings). Even in the absence of biological systems, this is exactly the type of molecule that would tend to create interfaces: monolayers on surfaces, bilayers in aqueous environments, and stable boundary films around droplets.

The boron spirocyclic motif is particularly interesting for abiotic chemistry because it pushes the boron–diol interaction away from “loosely exchanging complexes” and toward something that can behave like a more persistent conjugate (still reversible in principle, but less floppy). Boron binding to diols is already favored in alkaline conditions; when we build two cyclic boron–diol rings (dioxaborolane/dioxaborole-type motifs) and then fuse them through a spiro center, we create a headgroup that is (a) more rigid, (b) less conformationally random, and (c) likely to have a more defined hydration shell. In an abiotic setting, that matters because rigidity can translate into more reproducible membrane packing, less dependence on the exact transient conformation of the sugar, and potentially a higher “residence time” of the sugar at the interface. In other words, it’s a step toward headgroups that behave like designed surfactants, even though no biology is involved.

From a self-assembly standpoint, the molecule is essentially a glycolipid analog—but with boron providing the “glycosidic” linkage logic. Two octadecyl chains almost always bias an amphiphile toward bilayers and films rather than simple spherical micelles, because the hydrophobic volume is large and the molecular shape approaches cylindrical packing. The headgroup here is not small: it contains multiple oxygens, a hydroxymethyl substituent, and at least one remaining hydroxyl; plus boron itself can contribute charge or strong polarization depending on pH/counterion. That headgroup should be strongly hydrated and likely to generate a robust hydration layer at the interface. The likely outcome in water is not just aggregation, but membrane-like aggregation: lamellae, vesicles, and surface-supported films—especially in mixtures where some components are simpler amphiphiles (fatty acids, monoethers, alcohols) that can “fill in” packing frustrations.

The pH dependence remains central, but its character changes. In the simpler sugar–borate–polyol system, headgroup conjugation can be highly dynamic. In a spiro-borate motif, the headgroup may behave more like a pH-gated switch: stable in alkaline regimes where borate/boronate character is favored, but prone to weakening or rearrangement as conditions shift toward neutral or acidic. For abiotic settings, this can be a feature rather than a limitation: environmental cycling (wet–dry cycles, geothermal gradients, alkaline seepage into more neutral water) can repeatedly assemble and disassemble films. Such cycles are widely considered beneficial for prebiotic concentration and selection, as repeated assembly can concentrate solutes at interfaces and expose them to catalytic surfaces.

This molecule also has potential as an interfacial organizer of sugars. One of the central challenges in prebiotic chemistry is not only generating sugars, but stabilizing them in useful forms and preventing their diffusion or degradation. A boron-locked sugar headgroup anchored to a hydrophobic domain could act as a surface stabilizer, maintaining sugar functionality at the boundary of an aggregate where microenvironments differ significantly from bulk water (local pH, dielectric constant, water activity). Even if the bulk aqueous phase is unfavorable for free sugars, an interfacial sugar–boron headgroup could experience altered degradation kinetics and shifted equilibria among sugar forms. This provides a plausible route to surface enrichment of carbohydrate functionality prior to enzymatic systems—effectively a form of “carbohydrate display” on primitive membranes.

Another abiotic aspect is mineral surface compatibility. Highly oxygenated borate/sugar headgroups can form hydrogen bonds and coordinate with hydroxylated mineral surfaces (silicates, clays, metal oxyhydroxides). Double-tailed lipids, in turn, readily form supported bilayers and adsorbed monolayers. Together, these features suggest a model for abiotic surface films capable of coating mineral grains or lining pore structures in rock. In prebiotic scenarios, mineral pores and surfaces are often invoked as natural reactors; a surface-bound amphiphilic film can create a semi-permeable boundary and stabilize microcompartments while simultaneously presenting a sugar/boron-rich interface that may influence local chemistry (e.g., concentrating polyols, binding other diol-containing species, and altering ion distributions).

A final and important implication is that this type of structure suggests a plausible pathway to chemical selection without genetics. If boron-linked sugar headgroups form more stable interfacial amphiphiles than alternatives, they will be preferentially enriched in films and vesicles due to their greater persistence and assembly strength. If different sugars or polyols compete for boron binding, the system can effectively “select” the strongest binders through equilibrium and kinetic processes, enriching specific headgroups at interfaces. This enrichment then influences morphology—curvature, permeability, and film rigidity—which in turn affects which solutes are concentrated near or within the assembly. Such feedback loops resemble primitive selection pressures: not Darwinian replication, but physicochemical “survival of the stablest.”

The biggest caveat (and also a research opportunity) is that real-world behavior will depend on ionic strength, counterions, water activity, and mixture composition. Highly hydrated or charged headgroups can repel each other and increase curvature unless salts screen the repulsion; mixtures with simpler amphiphiles can stabilize bilayers; and drying/rehydration cycles can lock structures into different morphologies. However, this is precisely why the structure is interesting for abiotic chemistry: it is not a fragile “one-condition molecule,” but rather a candidate for environmentally responsive self-assembly, where pH and salts tune whether monolayers, vesicles, multilamellar stacks, or surface coatings are formed.

The spiro-boron designation may appear complex, but the underlying structure is conceptually elegant: it is a prebiotically “programmed” glycolipid analog—a molecule that combines (i) two long hydrophobic tails that strongly favor self-assembly with (ii) a sugar-derived, boron-locked polar headgroup that is both highly hydrated and structurally preorganized. If such a molecule can form under abiotic conditions, even in modest yields, it could function as a “keystone amphiphile”: not necessarily the most abundant component, but one that disproportionately influences the formation of stable interfaces, films, and membrane-like compartments.

The most important feature is the spiro-borate architecture itself. Ordinary borate–diol complexes can be transient, forming and dissociating in equilibrium, with sugars only intermittently associated with the boron center. A spirocyclic arrangement—two cyclic boron–diol rings sharing a central spiro junction—introduces a fundamentally different regime: a headgroup that is rigid, multidentate, and low-entropy. In soft-matter chemistry, rigidity is not merely structural; it encodes information. Rigid headgroups pack more predictably, generate reproducible interfacial hydration layers, and maintain defined geometries under conditions where flexible complexes would disperse into statistical disorder. In a prebiotic environment—mixed, dilute, and noisy—any motif that enhances reproducibility and persistence becomes a potential organizing principle.

The addition of two octadecyl ether chains further amplifies this effect. Two C18 tails provide a strong hydrophobic driving force for the formation of bilayers, vesicles, lamellar films, and surface coatings. Single-tailed amphiphiles tend to form micelles and highly curved aggregates, whereas double-tailed amphiphiles naturally favor membrane formation. This is why modern biological membranes rely on such structures. The molecule described here inherits this fundamental physical principle without requiring enzymatic synthesis or phosphate chemistry, effectively representing a membrane-forming system that could exist prior to biological evolution—provided that the necessary chemistry can occur.

The headgroup chemistry adds another layer of functionality. A boron–sugar headgroup is not a passive polar entity but a selective diol-binding module. In a chemically diverse prebiotic environment containing multiple polyols and sugars, boron exhibits selectivity for particular diol geometries and ring conformations. As a result, membrane surfaces could become sites of selective enrichment, where certain sugars accumulate preferentially due to stronger binding interactions. This represents a plausible mechanism for chemical selection without replication: no genetic system is required—only equilibrium processes and binding specificity that favor certain molecular motifs over others.

This leads to a broader implication: membranes may have originated as active selectors rather than passive barriers. A vesicle or surface film composed of such amphiphiles would present a dense array of oxygen-containing functional groups (ether linkages, hydroxyls, hydroxymethyl groups, and borate oxygens). This interface would structure water, bind cations, and create localized microenvironments with distinct polarity and water activity compared to the bulk solution. In prebiotic systems, such effects are critical, as reaction networks are often constrained not by formation pathways but by persistence and concentration. A sugar–borate headgroup anchored at a membrane interface could localize carbohydrate chemistry to a two-dimensional surface, effectively increasing local concentrations and enhancing interactions with catalytic minerals or metal ions. In this way, the membrane becomes a platform for concentration, organization, and templating of chemical processes.

The pH dependence transforms this system from a static lipid into an environmentally switchable assembly. Borate–diol interactions strengthen under alkaline conditions and weaken as conditions shift toward neutral or acidic regimes. This means the amphiphile can participate in a primitive form of environmental cycling: in alkaline niches (e.g., alkaline vents, evaporative ponds with elevated pH, carbonate-rich environments), sugar coordination is favored and the headgroup becomes maximally hydrated and structured; in more neutral waters, sugar attachment can relax or exchange. This creates a prebiotic analog of regulation, in which assembly, surface decoration, and permeability vary with environmental conditions, allowing compartments to “breathe” chemically as conditions fluctuate.

This is where the structure becomes especially compelling for abiotic chemistry: it could plausibly form films on mineral surfaces, coat grains, line pores, and stabilize droplets. Mineral surfaces are typically hydroxylated and capable of extensive hydrogen bonding. The headgroup is an oxygen-rich, boron-containing unit that can interact strongly with such surfaces, while the hydrophobic tails pack together to form a coherent layer. This combination provides a clear route to supported monolayers and bilayers. In porous rock, such films could generate semi-confined microreactors: thin membranes lining pores, concentrating sugars and polyols at boundaries while still permitting selective exchange of small molecules. In this view, the earliest “protocells” may not have been free-floating vesicles but rather membrane-coated pores or mineral interfaces. This molecule is well-suited to that scenario [76,77,78,79].

Even the question of film formation extends beyond surface tension to evolutionary relevance. Amphiphiles that form stable films under shear, dilution, or salt stress will persist longer in dynamic environments. Double-tailed lipids already exhibit this property; a rigid, boron-locked headgroup may enhance it further by enabling consistent packing and a durable hydration layer. If the amphiphile forms monolayers at air–water or solid–water interfaces, it could stabilize boundaries in environments that naturally generate interfaces: tidal flats, bubbles, foams, aerosols, porous rocks, droplets, and coacervate surfaces. Interfaces provide critical leverage for prebiotic chemistry, whereas bulk aqueous environments are often too dilute. A molecule that stabilizes such interfaces is therefore disproportionately important, even if present in low abundance [79,80,81,82].

These considerations lead to concrete, testable predictions that elevate the concept from speculative to experimentally tractable. Under mildly alkaline conditions with moderate salinity, the molecule should favor lamellar phases and vesicles, forming stable bilayer assemblies. At higher pH and lower salinity, increased headgroup charge and hydration should shift the system toward smaller vesicles or higher-curvature aggregates unless ionic strength screens repulsion. On silica or clay surfaces, strong adsorption and spontaneous formation of supported films are expected. In mixtures with other prebiotic amphiphiles (e.g., fatty acids, monoethers), the molecule should act as a bilayer stabilizer, lowering the threshold for vesicle formation and broadening the stability window. If these predictions are confirmed, this system represents not only a plausible molecular structure but also a viable mechanism by which abiotic chemistry could generate amphiphiles capable of forming membranes while selectively recruiting sugar-like functionality to membrane interfaces [83,84,85,86,87].

Thus, the potential of this structure lies not merely in the fact that it “could exist,” but in its role as a bridge molecule: a chemical bridge between polyol/sugar chemistry and membrane physics, and an evolutionary bridge between simple, fragile fatty-acid vesicles and the robust, functional membranes that life ultimately standardized. In a single molecule, three of the most challenging prebiotic problems—compartmentalization, concentration, and selectivity—begin to converge at the same interface [1,2,20,35].

One can connect the 3,4-bis(octadecyloxy)butane-1,2-diol–borate–ribose “abiotic membrane subunit” to archaeal lipids by treating it as a chemical ancestor in function, rather than a direct biosynthetic precursor in structure. This conjugate already contains two defining features of archaeal membranes: ether-linked hydrophobic chains and a highly polar headgroup capable of strong interfacial organization. Modern Archaea predominantly utilize ether lipids (e.g., archaeol diethers and GDGT tetraethers), which provide exceptional stability under extreme conditions [11,23,25,26,88,89]. In this system, the ether linkages to long C18 chains confer similar hydrolytic robustness (ethers are more resistant than esters), while the borate–sugar headgroup offers an abiotic pathway to functionality analogous to glycolipids—highly polar, hydrogen-bond-rich, and capable of recruiting and retaining carbohydrates at interfaces.

A coherent evolutionary narrative can thus be proposed: abiotic “polyol ether lipids” → early biological ether lipids → archaeal specialization. In prebiotic mixtures, polyols (including tetritols) capable of forming double-tailed ethers would have been particularly effective at assembling persistent bilayers and films [2]. With the emergence of primitive metabolism, biological systems likely converged on a smaller set of membrane backbones and stereochemistries—most notably reflected in the “lipid divide,” where Archaea construct membranes from ether-linked isoprenoids on glycerol-1-phosphate, while Bacteria and Eukarya rely on ester-linked fatty acids on glycerol-3-phosphate. Tetritol-based abiotic amphiphiles need not be direct biochemical precursors of archaeol [23,25]; rather, they can be understood as functional predecessors, demonstrating how ether-based membranes and complex headgroups could exist prior to the enzymatic pathways that later fixed archaeal lipid architecture.

The connection becomes particularly compelling at the level of membrane physics under environmental stress. Archaea regulate the ratio and cyclization of diether and tetraether lipids to maintain membrane integrity across extremes of pH and temperature. The present system predicts an analogous, but purely abiotic, mode of “adaptation”: pH governs borate speciation and thereby modulates headgroup size, charge, and hydration; ionic strength screens electrostatic repulsion; sugars compete for binding; and membrane morphology shifts accordingly (lamellae, smaller vesicles, or mixed phases) [2,90,91,92]. In this sense, processes that Archaea achieve genetically through lipid remodeling could, in an abiotic borate–diol system, arise from physicochemical equilibria and self-assembly—providing a plausible bridge from chemistry to biology.

Boron minerals enter this framework not simply as passive reservoirs of boron, but as geochemical selectors and stabilizers that can shape the prebiotic carbohydrate pool. Two roles are especially relevant [93,94,95]. First, borate released from boron-bearing minerals can participate in carbohydrate formation networks by binding to cis/vicinal diols, thereby altering reaction pathways and product distributions under formose-like conditions. Second, once sugars form, borate complexation can protect specific diol-rich conformations from rapid degradation, effectively introducing a selective stabilization step. The key experimental observation is that borate binds strongly to cis/vicinal diols and can stabilize ribose relative to competing aldopentoses, consistent with the classic result that “borate minerals stabilize ribose.” This stabilization is best understood as a conditional, environment-dependent effect: borate–diol interactions are modulated by pH, ionic strength, and water activity, so ribose enrichment is expected to occur within a bounded physicochemical window rather than universally. Subsequent work has extended this concept beyond a single “ribose protection” experiment by exploring mineral-guided cycles that generate and stabilize pentoses, with borate playing a binding and stabilizing role across repeated reaction–sequestration steps [30,31,65,96].

**Figure 3:**
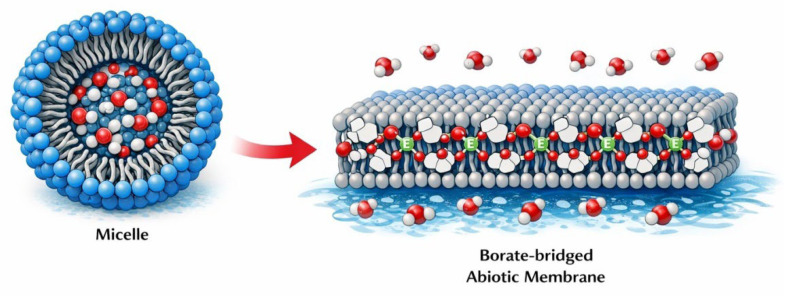
Schematic illustration of the structural transformation from a micelle to an abiotic membrane driven by the formation of borate-bridged amphiphile–carbohydrate conjugates. The membrane is stabilized through reversible borate ester linkages with the low-molecular-weight polyol, butane-1,2,3,4-tetraol, enabling the organization of amphiphilic molecules into a bilayer architecture reminiscent of primitive protocell membranes. Created in Adobe Illustrator Pro and Adobe Photoshop Elements 8.5.

From a membrane perspective, this framework is significant because it reframes boron minerals (often discussed within the colemanite/ulexite/kernite family) as environmental reactors that couple bulk-phase carbohydrate chemistry to interfacial processes. Specifically, such minerals can (1) supply borate to solution under appropriate conditions and (2) bias prebiotic chemistry toward diol-containing species—polyols and pentoses—that become preferred ligands for boron-mediated capture at interfaces (Figure 3). In other words, boron-bearing environments may enrich precisely the class of molecules (vicinal-diol substrates) that can be reversibly incorporated into borate-bridged headgroups on amphiphilic scaffolds. This provides a coherent bridge between “making sugars” and “retaining sugars in chemically active locations.” If borate-rich environments sustain a pool of diol-rich carbohydrates (including ribose-like motifs), and amphiphilic polyols present vicinal diols at hydrated interfaces, then boron can function as a reversible coupler that localizes carbohydrate functionality at membrane-like surfaces without enzymatic mediation. In this scenario, the compartment boundary becomes an active chemical interface: sugars are not merely present in bulk solution, but are displayed, exchanged, and stabilized within a hydrated headgroup layer whose occupancy is regulated by borate speciation. This interface-capture concept aligns with broader origin-of-life models in which minerals and surfaces act as active reactors that concentrate, select, and retain key functional molecules [30,31,96].

When these elements are combined, a plausible abiotic membrane scenario emerges with a strong conceptual link to archaeal systems: borate minerals (or borate-rich waters) help sustain a pool of diol-rich carbohydrates—particularly ribose-like motifs—while tetritol-derived double-tailed ether lipids provide persistent bilayer scaffolds. Borate then acts as a “coupling agent” that decorates membranes with sugars via diol binding, enabling surface-enriched carbohydrate chemistry in the absence of enzymes. The resulting assemblies behave as proto-glycolipid membranes: they form films on mineral surfaces, vesicles in aqueous environments, and dynamically exchange headgroup ligands depending on pH, ionic strength, and sugar composition [1,30,31]. This represents precisely the type of intermediate expected in a gradual evolutionary process: not yet genetically encoded, but already capable of the two essential membrane functions that enable life—compartmentalization and selective concentration at interfaces—which were later refined by biological systems (including Archaea) into standardized lipid architectures.

### 3.1. Qualitative Molecular Orbital Analysis

The butane-1,2,3,4-tetraol structure represents a borate-bridged amphiphile–carbohydrate conjugate in which a central boron atom adopts tetrahedral coordination with four oxygen atoms derived from carbohydrate hydroxyl groups (Table 3). The borate unit links two oxygen-rich fragments, while the molecule also contains a long hydrophobic amphiphilic chain, producing a strongly polarized amphiphilic architecture.

From an electronic standpoint, the most active region of the molecule is the borate–carbohydrate headgroup, which contains multiple oxygen atoms capable of donating electron density. In contrast, the hydrocarbon chain is electronically saturated and therefore contributes minimally to the frontier orbitals.

The highest occupied molecular orbital (HOMO) is expected to be localized primarily on the non-coordinated hydroxyl oxygen atoms and adjacent C–O bonds of the carbohydrate fragment, which represent the most electron-rich regions of the molecule. Oxygen atoms directly coordinated to boron contribute less strongly to the HOMO because the B–O bonds partially withdraw electron density from these atoms.

The lowest unoccupied molecular orbital (LUMO) is expected to be concentrated near the boron atom and neighboring oxygen-bearing carbons, reflecting the electron-deficient character of the borate center. Consequently, the frontier orbitals become spatially separated, with the borate–carbohydrate headgroup dominating electronic behavior while the hydrophobic chain functions primarily as a structural anchor.

The tetrahedral borate bridge also imposes geometric constraints that stabilize the relative orientation of the oxygen-containing fragments. This structural rigidity promotes a defined three-dimensional conformation and may facilitate self-assembly at aqueous interfaces, a property characteristic of amphiphilic molecules.

### 3.2. Borate-Bridged Phospholipids (BBPs)

**Figure 4:**
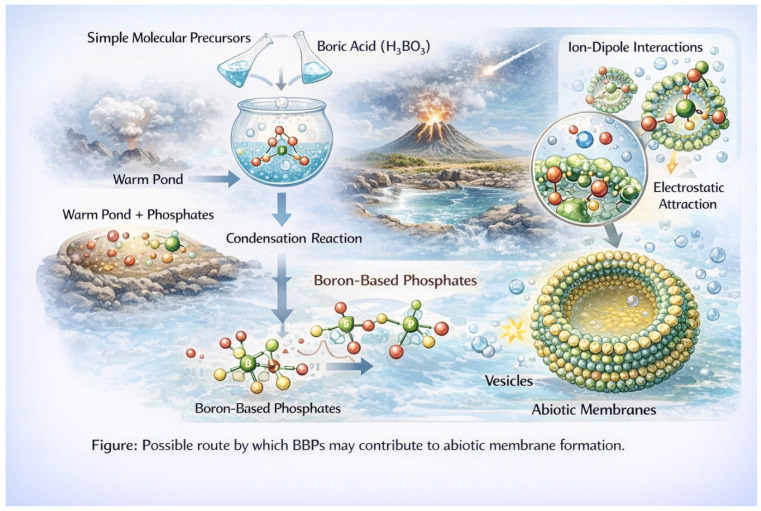
This illustration presents a proposed pathway for the formation of abiotic membranes mediated by boron-based phosphates (BBPs), starting from simple molecular precursors such as boric acid in a warm, prebiotic environment. Through condensation reactions and interactions with phosphates, BBPs are formed and subsequently engage in ion–dipole and electrostatic interactions that promote molecular self-assembly. These processes ultimately lead to the formation of vesicle-like structures, providing a plausible route toward primitive abiotic membranes. Created in Adobe Illustrator Pro and Adobe Photoshop Elements 8.5.

We extend this framework to propose a class of hypothetical compounds termed borate-bridged phospholipids (BBPs, Figure 4). These species represent a transitional form between simple amphiphiles and biologically derived phospholipids [1,2,20].

In this model, butane-1,2,3,4-tetraol serves as a central scaffold. Its four hydroxyl groups enable: (i) Partial functionalization with hydrophobic chains (forming amphiphilic character); (ii) Retention of vicinal diol motifs for borate binding, and (iii) Linkage to phosphorylated carbohydrate units derived from ribose.

Under prebiotic conditions, ribose could undergo phosphorylation in interfacial environments, such as aqueous microdroplets or dehydrating films, yielding sugar phosphates. These phosphorylated sugars could then associate with polyol-based amphiphiles, forming proto-phospholipid headgroups.

In BBPs, borate would act as a reversible bridging agent, linking polyol and carbohydrate components within the headgroup region. The resulting structure would exhibit: (i) A hydrophobic domain composed of alkyl or proto-lipid chains; (ii) A hydrophilic domain consisting of polyol and sugar phosphate units; (iii) A borate-mediated network stabilizing the interfacial region.

Importantly, BBPs are not expected to be discrete, uniform molecules, but rather ensembles of related species participating in a dynamic equilibrium.

### 3.3. Evolutionary Implications

BBPs may represent a plausible intermediate stage in membrane evolution, bridging the gap between: (i) Simple fatty acid vesicles, which are readily formed but environmentally fragile; and (ii) Fully developed phospholipid membranes, which require more complex synthetic pathways.

The incorporation of phosphate into amphiphilic systems confers increased stability under a range of conditions, including elevated temperatures and ionic strengths. Meanwhile, borate-mediated cross-linking could provide additional structural integrity in fluctuating environments.

Thus, BBPs could have functioned as adaptive, transitional membrane systems, capable of surviving in chemically harsh niches such as geothermal fields, while also participating in the emergence of phosphorylation-dependent chemistry.

### 3.4. Limitations and Experimental Outlook

Despite their conceptual appeal, several challenges remain: (i) The prebiotic synthesis pathways leading to BBP components are not yet fully established; (ii) Borate esters are reversible and sensitive to environmental conditions.

The integration of hydrophobic, polyol, and phosphorylated components into stable assemblies has not been experimentally demonstrated

## 4. Pentane-1,2,3,4,5-Pentaol Scaffold and Its Properties

This pentitol-based conjugate is a logical “next rung” in the same evolutionary ladder we’ve been building, because moving from tetritol to pentane-1,2,3,4,5-pentaol (a pentitol**)** does two things at once: it increases the valency of hydroxyl chemistry (more diol sites, more borate-binding options) and it increases the architectural degrees of freedom for building *very large, very hydrated headgroups* while still anchoring *multiple* hydrophobic chains [1]. If mathematical model predicts ~ 6.7% pentitols among abiotic polyols [1], that is not “rare”; it’s high enough that pentitol-derived amphiphiles could be a consistent minority component that strongly influences membrane behavior, especially because highly functional amphiphiles can dominate interfacial phenomena even at low mole fractions.

**Figure 5:**
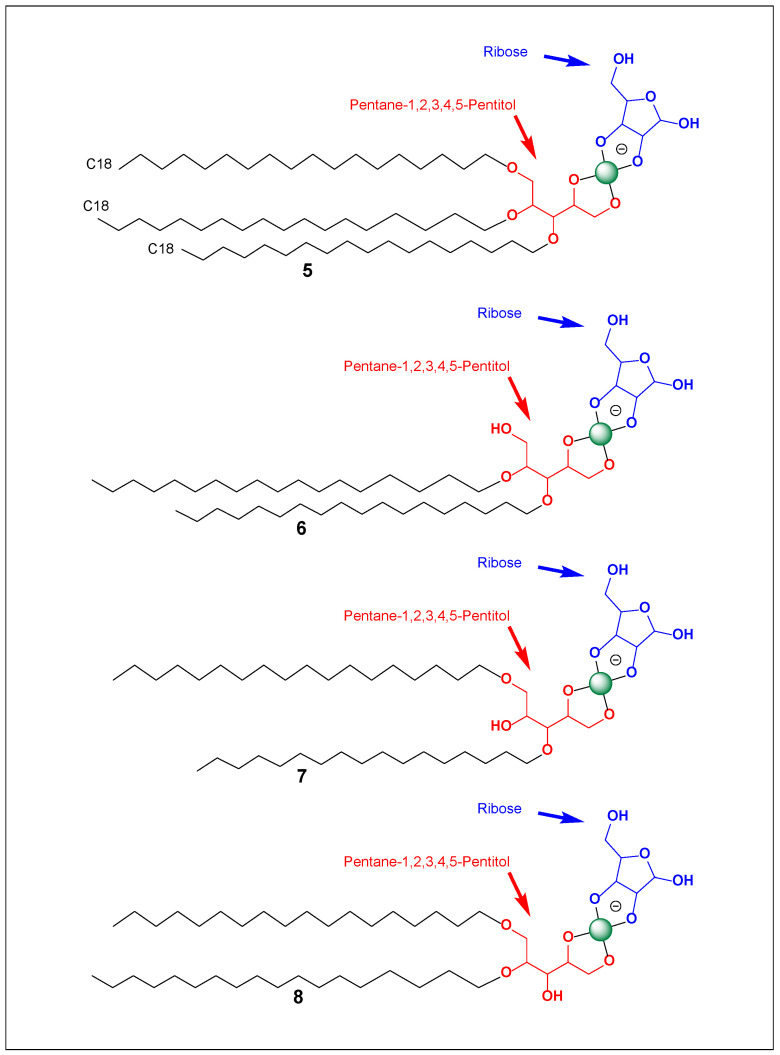
Structure of a borate-bridged amphiphile–carbohydrate conjugate based on the low-molecular-weight polyol, Pentane-1,2,3,4,5-pentaol. Isomers lacking one tail and one free hydroxyl group are shown; however, two hydrophobic tails remain present, allowing comparison with the corresponding tetritol-based borate-bridged amphiphile–carbohydrate conjugate. The additional hydroxyl group in the pentitol scaffold increases the number of possible borate coordination sites and enhances headgroup versatility. This structural difference may influence headgroup size, hydration, and crosslinking potential, thereby modulating self-assembly behavior and membrane mechanical properties relative to the tetritol-derived analogue.

Fundamentally, the named structure is best understood as a multi-tail amphiphile whose headgroup consists of a boron-locked sugar/polyol system. The fragment “tetrahydrospiro[[1,3,2]dioxaborolane-2,2′-furo[3,4-d][1,3,2]dioxaborol]-2-uide” indicates that the polar region is not a loose borate complex but a rigid, spiro-fused borate/boronate architecture—a headgroup that behaves as a preorganized, strongly hydrated “cap.” On the hydrophobic side, the molecule contains more than just two tails: it includes 1,2-bis(octadecyloxy) and one nonadecyloxy substituent, i.e., a three-chain hydrophobic domain (5, Figure 5). This configuration is structurally closer to membrane-anchoring lipids than to typical surfactants. In membrane physics terms, three chains increase the hydrophobic volume sufficiently that the system will strongly favor low-curvature aggregates—thick bilayers, multilamellar stacks, and robust surface films—unless the headgroup becomes sufficiently large or highly charged to induce curvature.

A pentitol scaffold specifically increases the likelihood of forming multiple borate rings and/or multipoint attachment because it contains several overlapping vicinal diol pairs (1,2; 2,3; 3,4; 4,5; Figure 3). This enhances “combinatorial binding”: boron can coordinate with the most favorable diol geometry available, thereby biasing which conformers dominate at the interface. This introduces a strong molecular selection step even prior to biological evolution: among many polyols, those presenting optimal diol geometries in a given microenvironment will form more persistent borate-linked headgroups and will be preferentially enriched in self-assembled phases. Pentitols, by offering more diol binding options than tetritols, can exhibit higher effective affinity for borate under alkaline conditions and can maintain binding across fluctuating environments, as alternative diol sites may compensate when others become unfavorable.

The spiro-boron headgroup also has important physical consequences: it promotes headgroup rigidity and persistent hydration. Flexible polyol headgroups can collapse, interpenetrate, and fluctuate, leading to highly variable prebiotic assemblies. In contrast, a rigid spiro architecture reduces this variability by establishing a consistent headgroup cross-section, maintaining a reproducible hydration shell, and supporting lateral organization through hydrogen-bonding networks among adjacent headgroups. In mixed amphiphile systems, these properties often promote domain formation—localized regions where rigid-headgroup amphiphiles cluster, stiffen the interface, and reduce permeability, even at modest concentrations. This behavior parallels how minority components can regulate membrane properties in modern systems (e.g., cholesterol-like effects), although here the organizing element arises from boron-locked polyol/sugar chemistry.

Because the conjugate contains three long alkyl chains, it is likely to behave less like a typical bilayer-forming lipid and more like a bilayer stabilizer, condensing agent, or even a promoter of non-lamellar phases, depending on the effective headgroup size. Three-chain amphiphiles generally increase membrane thickness, reduce water penetration, and enhance resistance to disruption by salinity, temperature fluctuations, and mechanical stress.

However, if the headgroup becomes excessively large and highly hydrated (as can occur with borate–sugar–polyol systems under strongly alkaline conditions), it may induce curvature frustration and promote bicontinuous or other complex morphologies in concentrated systems. Thus, this pentitol conjugate is best categorized as a “strong morphology modulator”: it can stabilize lamellar structures or drive transitions to non-lamellar phases depending on pH, ionic strength, and the presence of other amphiphilic components.

From an evolutionary chemistry perspective, the most important role of a ~6.7% pentitol fraction [1] is that it could function as a minority component that extends the stability window of primitive membranes. Early amphiphile assemblies are notoriously sensitive to divalent cations, salinity, and pH. A multichain amphiphile with a rigid, highly hydrated headgroup can reduce these sensitivities by providing (i) strong hydrophobic cohesion and (ii) a thick, highly hydrated interfacial layer that resists collapse. At the same time, borate dependence introduces a “chemical switch”: in alkaline niches (e.g., serpentinization systems, carbonate-rich ponds), these headgroups would be favored and membrane stabilization enhanced; in neutral or acidic conditions, headgroup binding may relax, potentially increasing permeability or enabling structural remodeling. This type of environment-coupled stability is precisely what is expected for abiotic systems evolving toward biological robustness.

The fundamental point is that pentitol-derived conjugates such as the one described are not merely larger analogs of tetritol systems—they introduce a qualitatively new capability: higher valency and redundancy in diol binding, which makes boron-mediated sugar capture and headgroup persistence more reliable across fluctuating conditions. In a prebiotic environment characterized by gradients and cycles, redundancy functions as robustness. It allows the membrane to maintain interfacial organization without relying on a single binding geometry. Thus, even at ~6.7% [1], pentitol subunits could act as structural organizers, stabilizers, and selectors—supporting membrane persistence under challenging conditions while concentrating sugars and polyols at interfaces, prior to the emergence of enzymatic lipid biosynthesis.

Extending this concept further, a key experimental prediction is that introducing a small mole fraction of this pentitol tri-tail conjugate into a “base membrane” composed of simpler diol or tetritol ether lipids should produce measurable changes in physical properties: reduced critical aggregation concentration, decreased permeability, increased bending modulus, thicker membrane films, and enhanced adsorption to mineral surfaces. The magnitude of these effects should increase with pH (as borate coordination strengthens) and decrease in the presence of competing diols that displace the sugar headgroup.

### 4.1. Physical Properties of Borate-Bridged Amphiphile–Carbohydrate Conjugate Built on Pentane-1,2,3,4,5-Pentitol (Two Tails)

Although we can’t “measure” these properties without experiments, we *can* make defensible, order-of-magnitude estimates for a borate-bridged amphiphile–carbohydrate conjugate built on pentane-1,2,3,4,5-pentitol (6, 7, and 8), especially if we assume the common case we’ve been discussing: two long ether tails on the pentitol scaffold and a borate bridge to ribose (a glycolipid-like headgroup).

#### 4.1.1. Molar Mass and Size

A useful baseline is the diether pentitol before sugar/borate binding. If two octadecyl ether chains are installed on a pentitol (C_5_H_12_O_5_), we effectively add 2 × C_18_H_37_ and remove 2H (for ether formation), giving an approximate diether pentitol formula near C_41_H_84_O_5_, corresponding to a molar mass of about ≈ 644 g/mol (carbon: 41 × 12.01 ≈ 492.4; hydrogen: 84 × 1.008 ≈ 84.7; oxygen: 5 × 16 = 80 → total ≈ 657; depending on the exact substitution pattern and whether one OH is lost/modified, we’ll typically land in the ~630–660 g/mol range). Adding ribose (~150 g/mol) and boron (~10.8 g/mol) and accounting for the borate ester condensation (net loss of ~1–2 H depending on charge/protonation state) gives a practical conjugate mass of roughly ~790–820 g/mol for the “one ribose + one boron” species. If a second sugar binds (possible for higher-valency scaffolds), add another ~150 g/mol per sugar.

#### 4.1.2. pH-Dependent Stability and Speciation

The conjugate’s persistence is controlled mainly by boron speciation and diol deprotonation. Below roughly pH 8–9, borate–diol complexation is weaker, and the system will spend more time in “unbridged” states (pentitol diether with free OH, plus free sugar). As pH rises toward ~9–10, the borate fraction increases sharply and cyclic borate esters become much more favorable; this is the regime where the bridged amphiphile–ribose headgroup should dominate. At still higher pH, binding can strengthen further, but the headgroup also tends to become more highly hydrated and more anionic in effective behavior, which can increase electrostatic repulsion between neighboring headgroups in aggregates unless ionic strength screens it. In short, maximum conjugate fraction and longest residence time at the membrane/interface are expected in mildly to strongly alkaline conditions, and salt will stabilize assemblies by screening headgroup repulsion.

#### 4.1.3. Self-Assembly Tendency, Packing Parameter, and What Structures Form

With two tails, a pentitol-based conjugate behaves much more like a bilayer lipid than like a surfactant. We can estimate the packing tendency with the critical packing parameter *p* = *v*/(*a*_0_*l*). Two C18 tails give a combined hydrophobic volume *v* on the order of ~1000 Å^3^ and an extended tail length lll around ~24–25 Å. The key variable is the effective headgroup area a_0_. For a compact pentitol headgroup (weak borate/sugar loading), a0a_0a0 might be ~60–80 Å^2^, giving *p* ≈ 0.5–0.7P\approx 0.5–0.7P ≈ 0.5–0.7, which strongly favors bilayers/vesicles/lamellae. When borate bridges ribose, a0a_0a0 can swell to ~90–130 Å**^2^**(hydration + sugar bulk + charge), pushing PPP down toward ~0.3–0.5; that range still allows bilayers, but increases the likelihood of smaller vesicles, higher curvature, and mixed phases unless ionic strength is sufficient. This leads to a clear prediction: pentitol–borate–ribose diethers should form bilayers readily, but vesicle size and morphology should be highly tunable by pH and salt, with higher pH (more sugar-borate occupancy) tending to increase curvature unless screened.

#### 4.1.4. Interfacial Properties and Film Formation

Even without knowing exact CMC/CAC values, we can predict *very strong* film-forming behavior. Two long chains plus a highly oxygenated headgroup make the molecule extremely surface-active at air–water, oil–water, and especially mineral–water interfaces (silica/clays). The borate–sugar headgroup increases hydrogen bonding and hydration, which tends to produce stable, elastic interfacial films (good at coating surfaces and stabilizing droplets). If the conjugate is partially anionic at alkaline pH, it may show a substantial negative zeta potential (often tens of mV in magnitude in analogous anionic surfactant systems), which helps keep vesicles/micelles colloidally stable by electrostatic repulsion; adding salt reduces the magnitude but can improve bilayer packing.

#### 4.1.5. Mechanical and Thermal Expectations

Compared to a tetritol analogue, the pentitol scaffold adds one extra OH and therefore increases headgroup hydration and binding options for borate. Mechanically, that generally means stiffer surfaces when borate binding is strong (because headgroups become more structured and can interact laterally), potentially increasing bending modulus and lowering permeability. At the same time, higher hydration can increase area per lipid and soften packing unless salt screens repulsion—so the mechanical effect is condition-dependent: in alkaline + moderate salt, expect robust, relatively stiff bilayers; in alkaline + low salt, expect more expanded, curvature-stressed membranes. If tails are saturated (e.g., C18:0), chain melting/ordering transitions can occur at higher temperatures than for unsaturated tails, meaning membrane rigidity will be strongly tail-chemistry dependent (selachyl-like unsaturation yields more fluid assemblies).

### 4.2. Physical Properties of Borate-Bridged Amphiphile–Carbohydrate Conjugate Built on Pentane-1,2,3,4,5-Pentitol (Three Tails)

**Figure 6:**
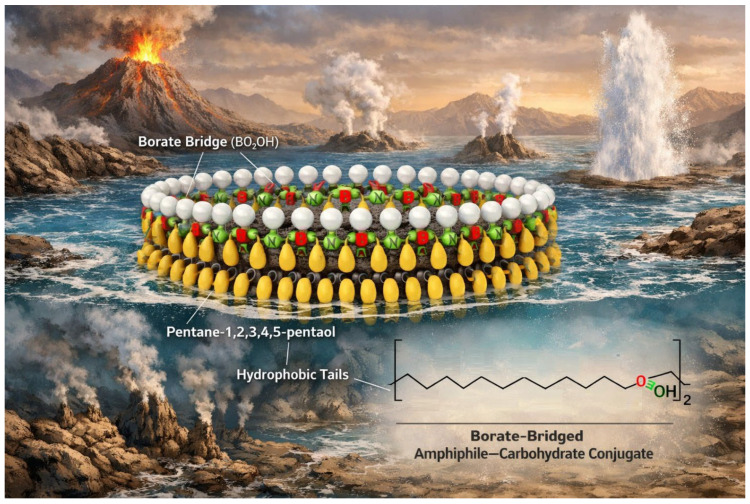
Conceptual illustration of a bilayer abiotic membrane composed of borate-bridged amphiphile–carbohydrate conjugates derived from the low-molecular-weight polyol pentane-1,2,3,4,5-pentaol. The structure features hydrophilic polyol head groups cross-linked by borate esters and hydrophobic tails forming a stable bilayer, plausibly arising under prebiotic conditions in hydrothermal, volcanic, or geyser-associated environments on early Earth. Created in Adobe Illustrator Pro and Adobe Photoshop Elements 8.5.

A borate-bridged amphiphile–carbohydrate conjugate built on a pentane-1,2,3,4,5-pentitol backbone and carrying three C18 ether tails (5, Figure 5) is, in physical terms, a highly hydrophobic, strongly surface-active, low-CMC “super-amphiphile” whose headgroup chemistry is pH-gated by borate–diol complexation (see Table 4). Structurally, this system consists of a pentitol scaffold in which three hydroxyl groups are etherified (three octadecyl chains), while the remaining hydroxyls (typically two, depending on the substitution pattern) provide vicinal diols for borate binding; boron then bridges to a sugar diol (e.g., ribose), generating a bulky, highly hydrated headgroup. Because this conjugate is tri-tailed, it will not behave like a simple micelle-forming amphiphile, as seen in single-tail batyl/selachyl/chimyl systems; instead, it strongly favors low-curvature assemblies (lamellae, thick bilayers, multilamellar stacks) and, under certain hydration and ionic conditions, complex non-lamellar phases (bicontinuous/cubic or inverted structures) rather than small spherical micelles. The key “switch” resides in the headgroup: at lower pH, borate bridging is weaker and the headgroup is smaller and less charged, increasing effective hydrophobicity and promoting more condensed, less hydrated phases; at mildly to strongly alkaline pH, borate–diol binding is favored, the ribose-bearing headgroup expands and becomes more hydrated, and the assembly is stabilized in aqueous environments but becomes more sensitive to ionic strength due to increased electrostatic and headgroup repulsion (Figure 6).

We can estimate the molar mass to understand the scale of this system. A tri-C18 ether pentitol (three octadecyl chains attached to a C5 polyol) corresponds approximately to C59H120O5 (5), yielding a molecular weight of ~910 g/mol for the tri-tail scaffold alone. Upon formation of the borate–ribose conjugate, the total mass is approximately the sum of the scaffold, ribose, and a boron source, minus the water molecules eliminated during borate ester formation. Using ribose (~150 g/mol) and boric acid (~62 g/mol), and assuming elimination of ~2 water molecules (consistent with a bridging diester), the estimated molecular weight falls in the range of ~1080–1100 g/mol for the conjugate (the exact value depends on protonation state and whether boron retains hydroxyl substituents). This high molecular weight, combined with three saturated C18 chains, implies (i) extremely low monomer solubility, (ii) very low effective CMC/CAC (aggregation at very low concentrations), and (iii) strong adsorption and film formation at interfaces.

From a packing-parameter viewpoint, three C18 chains provide a combined hydrophobic volume on the order of ~1500 Å^3^ (three tails) and an extended chain length of ~24–25 Å. The decisive term is the effective headgroup area *a*_0_. With borate–ribose attached, *a*_0_ is likely large—often plausibly ~120–180 Å^2^ depending on pH, counterions, and hydration. Plugging into *p* = *v*/(*a*_0_*l*) gives p ≈ 1500/(120·24) = 0.52, p ≈ 1500/(120·24) = 0.52 (bilayer-favoring) down to p ≈ 1500/(180·24) = 0.35, p ≈ 1500/(180·24) = 0.35 (higher-curvature, more “expanded headgroup” behavior). That means this conjugate can sit near a bilayer/lamellar sweet spot when the borate–ribose headgroup is fully engaged and hydrated, but it can drift toward non-lamellar/inverted tendencies if (a) the headgroup is not fully “swollen” (lower pH, weaker borate binding) or (b) dehydration/charge screening collapses headgroup repulsion (high salt, low water activity). In practical terms, alkaline + moderate ionic strength should favor robust lamellae/vesicle-like assemblies and durable surface films; very high salt or partial headgroup collapse can promote tighter packing and possibly bicontinuous or inverted phases in concentrated systems.

Finally, the tri-tail (**5**) nature makes this conjugate especially potent for film formation and membrane mechanics. Expect strong, persistent adsorption onto hydrophilic minerals (silica/clays) and high surface elasticity because the three chains anchor tightly while the borate–ribose headgroup creates a thick hydration layer. Compared with a two-tail pentitol analog, a three-tail conjugate should yield lower permeability, higher bending modulus (stiffer membranes), thicker hydrophobic cores, and a stronger tendency toward multilamellarity—yet still retain environmental responsiveness because the borate bridge is pH-dependent and can exchange diol ligands. In abiotic-membrane framework, that combination is exactly what a “minority structural organizer” would do: even at relatively low abundance, tri-tail pentitol borate–sugar conjugates could stiffen and stabilize primitive films, broaden the stability window in salty environments, and create carbohydrate-decorated interfaces without enzymes.

## 5. Hexane-1,2,3,4,5,6-Hexitol Scaffold and Its Properties

**Figure 7:**
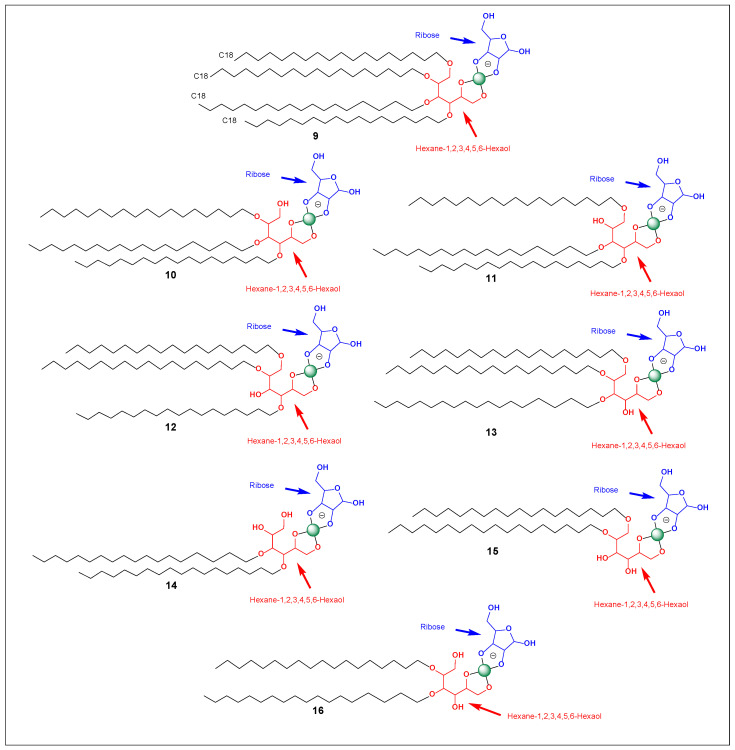
Structure of a borate-bridged amphiphile–carbohydrate conjugate based on the low-molecular-weight polyol hexane-1,2,3,4,5,6-hexitol. This conjugate can theoretically generate more than ten structural isomers, depending on the number and position of etherified hydroxyl groups and borate-mediated linkages. Shown are representative isomers lacking one or two hydrophobic tails and one or two free hydroxyl groups; however, variants bearing two or three alkyl chains remain amphiphilic and capable of self-assembly. The higher hydroxyl valency of the hexitol scaffold increases the number of possible borate coordination modes and enhances structural diversity at the headgroup region. In certain configurations, an additional ribose molecule may coordinate through borate bridging, further enlarging and hydrating the polar domain. Such increased hydrophilicity and multivalency may significantly influence aggregate morphology, membrane rigidity, and interfacial stability under varying pH and ionic conditions.

Moving to a hexane-1,2,3,4,5,6-hexitol scaffold changes the problem qualitatively, not just quantitatively. Even if model assigns only ~2.1% abundance [1] to hexitols in the abiotic polyol pool, that percentage is high enough for them to act as structurally influential minority components (Figure 7). In interfacial systems, a small fraction of highly functional amphiphiles can dominate phase behavior and mechanical properties. The reason is simple: valency scales nonlinearly—a molecule with six hydroxyls is not “twice as functional” as a triol; it is geometrically capable of an entirely different class of network and binding topologies.

From a structural standpoint, hexitol contains five adjacent C–C bonds and therefore multiple overlapping vicinal diol pairs (Figure 4): (1,2), (2,3), (3,4), (4,5), (5,6). This creates a dense combinatorial landscape for borate complexation. Boron can form cyclic esters with any favorable 1,2-diol geometry, and if multiple borate units are present, crosslinking between different diol pairs becomes possible. Compared to tetritol or pentitol, hexitol introduces a form of binding redundancy: if one diol geometry is disrupted by temperature, pH, or conformational fluctuation, another may remain accessible. That redundancy increases the persistence of borate–polyol complexes under fluctuating prebiotic conditions.

Now consider amphiphilicity. A hexitol backbone can, in principle, accommodate two, three, or even four hydrophobic tails, depending on how many hydroxyls are etherified. Each additional tail dramatically increases hydrophobic volume and shifts the packing parameter. With two tails, the molecule resembles a classical bilayer lipid. With three tails, it becomes a membrane-condensing agent that thickens and stiffens assemblies. With four tails, it approaches the behavior of tetraether-like architectures, conceptually similar to archaeal glycerol dialkyl glycerol tetraether lipids that span membranes and provide extreme stability. The theoretical possibility of four hydrophobic chains anchored to a single polar hexitol core means that this scaffold could generate amphiphiles with very low curvature preference, favoring robust lamellae, multilamellar stacks, or even quasi-monolayer-spanning membranes in confined geometries.

The headgroup side becomes even more interesting when ribose enters. With six hydroxyl groups, hexitol offers many potential attachment sites for borate-mediated sugar conjugation. Ribose itself contains vicinal diols capable of forming cyclic borate complexes. In a hexitol–borate–ribose system, several structural scenarios emerge: (1) one boron atom bridging a hexitol diol and a ribose diol; (2) multiple boron centers coordinating distinct diol pairs; (3) intramolecular spiro-borate motifs creating rigid, multidentate headgroups; (4) even limited crosslinking between adjacent amphiphiles if borate bridges span molecules. The combinatorial diversity of possible linkages means that the headgroup architecture can range from compact cyclic borates to extended, partially networked interfacial structures.

From a fundamental thermodynamic perspective, this introduces the possibility of headgroup networking at the membrane surface. Whereas tetritol systems may provide discrete borate–sugar headgroups, hexitol systems can support partial lateral crosslinking through shared borate coordination. Such networking would increase the bending modulus of the membrane, reduce permeability, and stabilize the assembly against salt or temperature fluctuations. In a prebiotic ocean with variable ionic strength, this would be advantageous. The membrane would not just be a hydrophobic barrier; it would be a semi-crosslinked, hydrated interfacial layer with emergent mechanical resilience.

At the same time, the large number of hydroxyl groups means that the headgroup is extremely hydrophilic and strongly solvated. If too many hydroxyls remain free (i.e., insufficient hydrophobic substitution), the molecule may fail to aggregate efficiently because the hydrophilic fraction dominates. Therefore, there is an optimal substitution regime. Two-tail hexitol amphiphiles likely favor bilayers. Three-tail systems could condense and stiffen membranes. Four-tail systems might promote quasi-monolayer or membrane-spanning arrangements. Thus, hexitol scaffolds introduce a tunable spectrum of membrane morphologies, depending on substitution pattern and environmental conditions.

In evolutionary terms, a 2.1% abundance [1] is not negligible. In heterogeneous prebiotic mixtures, even a small fraction of highly multivalent amphiphiles can act as structural nucleators—initiating lamellar phases, stabilizing vesicles, or anchoring membranes to mineral surfaces. Because hexitol derivatives can form multiple borate complexes simultaneously, they may also preferentially accumulate in alkaline, borate-rich niches. This would create spatial heterogeneity: certain environments would favor hexitol-rich membranes, while others would not. Such environmental sorting is a plausible mechanism for early chemical selection.

Most fundamentally, hexitol conjugates expand the evolutionary design space of membranes. They introduce high valency, redundancy, potential crosslinking, and multi-tail hydrophobic architectures into an abiotic setting. These features increase robustness, reduce reliance on a single binding geometry, and allow membranes to respond dynamically to pH and borate availability. Even if hexitols represent only a small statistical fraction of polyols, their structural versatility could make them disproportionately important in the transition from fragile amphiphile aggregates to persistent, evolution-capable protomembranes.

### 5.1. Baseline Chemistry That Matters for All Three

A reasoned, quantitative-leaning estimate for hexane-1,2,3,4,5,6-hexitol (C_6_H_14_O_6_)–based borate-bridged amphiphile–carbohydrate conjugates carrying 2, 3, or 4 C18 ether tails, and a comparison of which is “better” for forming abiotic membrane-like assemblies (Figure 7).

A hexitol headgroup is high-valency: it contains many overlapping vicinal diols (1,2; 2,3; 3,4; 4,5; 5,6), so under alkaline conditions it can bind borate strongly and redundantly. That means (i) borate bridging to ribose is easier to sustain across fluctuating conditions, and (ii) it can support more than one borate center and/or more than one sugar in principle. Physically, borate–ribose binding increases headgroup size, hydration, and often effective anionic character, which pushes toward higher curvature unless screened by salt, while increasing colloidal stability once assemblies form.

#### 5.1.1. Estimated Molar Mass (Order-of-Magnitude)

Assume C18 tails are installed as octadecyloxy (C_18_H_37_O–) ethers onto the hexitol and at least one ribose + borate is attached via borate bridging.

Hexitol di-C18 ether (2 tails, 14, 15, and 16): Approx. formula ~ C_42_H_88_O_6_ → MW ≈ ~665 g/mol ribose (~150) + boron (~11) and ester-condensation bookkeeping → ~810–850 g/mol total (typical “one ribose + one boron” conjugate)

Hexitol tri-C18 ether (3 tails, 10, 11, 12, and 13): Approx. formula ~ C_60_H_124_O_6_ → MW ≈ ~917 g/mol ribose + boron → ~1.06–1.12 kDa

Hexitol tetra-C18 ether (4 tail, 9): Approx. formula ~ C_78_H_160_O_6_ → MW ≈ ~1.17 kDa

ribose + boron → ~1.32–1.38 kDa

These are “backbone” values; real samples will exist as microheterogeneous families (different substitution positions, different borate hydration/protonation/counterions, sometimes 2 sugars).

#### 5.1.2. Packing/Shape and Dominant Morphology: 2 vs. 3 vs. 4 Tails

A fast way to compare assembly preference is the packing parameter logic: increasing tail number increases hydrophobic volume **v** strongly, while borate–ribose increases headgroup area **a_0_**. Hexitol’s ability to carry multiple borate/sugar attachments makes **a_0_** more “expandable” than tetritol/pentitol.

Two tails (2 × C18): “classic bilayer lipid behavior”

Best-fit morphology: bilayers, vesicles, lamellae over a broad condition range

Why: two tails usually produce near-cylindrical shape; hexitol + borate + ribose gives a large headgroup, but not so large that it *must* micellize.

pH/salt response: low pH/weak borate: headgroup smaller → more condensed bilayers alkaline pH/strong borate–ribose: headgroup expands/charged → smaller vesicles and higher curvature unless salt screens

Practical expectation: This is the most “membrane-like in water” option—most likely to give true vesicles rather than precipitates.

Three tails (3 × C18): “membrane condensing + film-forming amphiphile”

Best-fit morphology: thick lamellae, multilamellar vesicles, robust surface films, sometimes complex phases at higher concentration

Why: three tails greatly increase hydrophobic cohesion and lower permeability; the molecule can behave like a bilayer stabilizer/condensing agent.

pH/salt response:

alkaline + low salt: headgroup charge/hydration can drive curvature frustration → smaller vesicles or mixed lamellar/nonlamellar

alkaline + moderate salt: screening restores packing → very stable lamellae/MLVs

Practical expectation: Often the “best” choice for abiotic membranes that must survive salt/heat/shear, and for mineral-supported films. It may be less likely than 2 tails to form nice monodisperse vesicles, but more likely to form durable boundary layers.

Four tails (4 × C18): “too hydrophobic for free vesicles, but ultra-stable when confined”

Best-fit morphology: strongly condensed lamellar stacks, waxy phases, supported films, potentially inverted/nonlamellar phases in concentrated regimes

Why: four tails push hydrophobic volume so high that dispersion in bulk water becomes hard unless the headgroup is *very* expanded (multiple sugars/borates) and/or assisted by co-surfactants. This can behave more like a membrane-spanning/tetraether-like stabilizer in function (not structure-identical to archaeal tetraethers, but similarly “hard to disrupt”).

pH/salt response: alkaline + strong borate + high hydration: may become dispersible as stiff aggregates, high salt/dehydration: can crash out into dense phases

Practical expectation: Best as a minority “rigidity and barrier enhancer” in mixed membranes or as a surface-bound membrane coating in pores/minerals—not necessarily as the dominant lipid for free vesicles in open water.

#### 5.1.3. Predicted Mechanics and Permeability

2 tails: lowest bending modulus of the three (still substantial), highest fluidity, highest permeability (still low compared with fatty-acid vesicles if chains are saturated), best for dynamic membranes.

3 tails: higher bending modulus, lower permeability, thicker hydrophobe core, stronger resistance to salts and divalent ions; good “robust protomembrane.”

4 tails: highest stiffness, lowest permeability, strongest barrier; but also greatest risk of poor dispersibility and phase separation.

A key hexitol-specific point: because hexitol can support multiple borate binding modes, it can create headgroup networking at high pH/borate, which stiffens surfaces further—especially for 3- and 4-tail variants.

#### 5.1.4. Colloidal Stability and Solubility in Water

2 tails (14–16): likely to form stable dispersions (vesicles/lamellae) at relatively low concentration once above CAC; easiest to work with in water.

3 tails (10–13): very low CAC; stable assemblies form easily, but dispersions may be more polydisperse/multilamellar.

4 tail (9): lowest CAC but also lowest monomer solubility; strong tendency toward aggregation/precipitation unless headgroup is highly charged/hydrated and/or with other amphiphiles.

### 5.2. Which Is “Better” for Abiotic Membranes?

It depends on what we mean by “better,” so here are the clean conclusions: (i) Best for forming true vesicles in bulk water (protocell-like compartments): → 2 tails; (ii) Most reliable bilayer former; broadest workable window; (iii) Best for durability, surface films, and mineral/pore coatings (abiotic boundary layers): → 3 tails. Strong “membrane stabilizer,” likely to persist under harsh conditions; still amphiphilic enough to remain interface-active; (iii) Best as a minority component to harden and seal membranes (extreme stability), not as the bulk lipid: → 4 tails. Think “structural reinforcing agent” rather than primary membrane former.

If want a single “most prebiotically useful” choice across many plausible environments (alkaline + saline + mineral interfaces), I would pick 3 tails as the most powerful *organizer/stabilizer*, with 2 tails as the best *bulk-forming lipid* for compartments. The 4-tail version is fascinating, but likely requires mixtures or confinement to show its advantages without simply phase-separating.

## 6. Fundamental Comparison of Tetritol, Pentitol, and Hexitol Scaffolds

Below is a side-by-side, *fundamental* comparison of tetritol (C4 tetraol), pentitol (C5 pentaol), and hexitol (C6 hexaol) scaffolds as abiotic membrane subunits, focusing on (1) packing/shape ranges, (2) borate crosslinking potential, and (3) predicted membrane mechanics [1].

### 6.1. Tetritol Scaffold (Butane-1,2,3,4-Tetraol Derivatives)

A tetritol contains multiple vicinal diols but only a modest number of distinct binding “choices,” so its borate chemistry tends to be strong yet not excessively multivalent. In practice, this makes tetritol an effective “bridge scaffold”: it can accommodate two hydrophobic chains (as a diether) while still retaining one vicinal diol available for borate and sugar binding. This architecture typically produces two-tailed amphiphiles with headgroups that can expand under alkaline borate/sugar loading.

From a packing-parameter perspective, tetritol diethers span a useful range: with a compact headgroup (low borate/sugar occupancy), they reside in the bilayer/vesicle regime; with borate–sugar-induced headgroup expansion, they shift toward higher-curvature structures (smaller vesicles or mixed phases), but generally do not become “network formers” because headgroup valency remains limited. Mechanically, tetritol-based bilayers are expected to be moderately stiff (due to two hydrophobic tails), with stiffness and permeability strongly tunable by pH and ionic strength, as headgroup hydration and charge are environmentally responsive. The defining feature of tetritol systems is reversible functionality without excessive gelation: they can self-assemble into membranes that are stable yet remain dynamically adaptive.

### 6.2. Pentitol Scaffold (Pentane-1,2,3,4,5-Pentaol Derivatives)

Pentitols step up the chemistry by adding one more hydroxyl, and that change disproportionately increases headgroup possibilities because it creates more overlapping diol pairs and more conformational “solutions” for borate ring formation. Pentitol-based amphiphiles can still form clean two-tail lipids, but they also more readily accommodate three-tail variants (as in tri-tail example), which have major packing consequences: three tails strongly bias the molecule toward low curvature and high cohesion, often behaving like a membrane-condensing lipid that thickens bilayers and reduces permeability. At the same time, the headgroup can become larger under borate/sugar loading, so pentitol lipids can occupy both extremes: they can act as curvature generators when the headgroup dominates, or as curvature suppressors when hydrophobic volume dominates (three tails). Crosslinking potential is meaningfully higher than tetritol because pentitol can support multiple borate interactions or more persistent spiro-borate headgroups; this makes surface networking and domain formation more likely (even at modest mole fractions). Mechanically, pentitol-rich membranes are predicted to show higher bending modulus (stiffer), lower permeability, and stronger film formation on surfaces, but also an increased chance of phase separation (rigid headgroup domains) if borate crosslinking becomes extensive.

### 6.3. Hexitol Scaffold (Hexane-1,2,3,4,5,6-Hexaol Derivatives)

Hexitols represent a qualitative leap because their valency becomes sufficiently high for redundant binding and multipoint coordination to dominate behavior. With six hydroxyl groups, multiple vicinal diol pairs are available, allowing the formation of more than one borate ring per molecule; at sufficient borate availability, this can transform the headgroup region into a semi-networked layer rather than a collection of isolated headgroups. Hydrophobic substitution is highly flexible: a hexitol scaffold can plausibly support two, three, or four alkyl chains, enabling a wide range of packing behaviors. Two-tail hexitol derivatives may behave similarly to conventional bilayer-forming lipids but with larger, more hydrated headgroups; three-tail variants can act as strong condensing agents; and four-tail configurations conceptually approach tetraether-like membrane stabilizers that may significantly reduce permeability and enhance thermal robustness.

The primary limitation is the risk of “overfunctionality.” If too many hydroxyl groups remain unmodified and borate/sugar loading is high, the headgroup can become excessively hydrated and highly charged, leading to strong electrostatic repulsion and curvature frustration unless mitigated by ionic screening. At the same time, increased multivalency may promote crosslinking, potentially resulting in gel-like or partially solidified interfacial structures. Mechanically, membranes incorporating hexitol-based amphiphiles are expected to be among the stiffest and least permeable, with a strong tendency to form robust surface films; however, they are also the most susceptible to transitions into non-lamellar phases, rigid domains, or partial gelation depending on environmental conditions.

### 6.4. Packing Parameter Ranges in One Conceptual Line

Tetritol diethers: broad but centered around bilayers/vesicles, with pH/sugar pushing toward smaller curvature.

Pentitol di-/tri-tail: spans bilayers → very low curvature lamellae, with stronger ability to “lock” membranes and form rigid domains.

Hexitol (2–4 tails): spans almost everything from bilayers to ultra-stable lamellae and networked interfacial phases, depending on substitution and borate/sugar occupancy.

As hydroxyl valency rises, the headgroup becomes more capable of both (a) chemical recognition and (b) mechanical reinforcement, but also more capable of producing network effects that can either stabilize membranes or over-stiffen/gel them.

#### 6.4.1. Crosslinking Potential and What It Means Physically

Tetritol: mostly discrete complexes; crosslinking is limited, so assemblies remain fluid and adaptive.

Pentitol: moderate multi-point binding; increased likelihood of “patchy” crosslinking and domain formation.

Hexitol: high multi-point binding; can approach a percolating network at the interface under favorable pH/borate, turning the headgroup region into a mechanically active layer (stiffer, less permeable, more film-like).

In prebiotic terms, tetritol supports “responsive membranes,” pentitol supports “reinforced membranes,” and hexitol supports “membranes with surface scaffolding.”

#### 6.4.2. Predicted Membrane Mechanics as a Gradient

If we imagine adding each scaffold into a mixed protomembrane: (i) Tetritol fraction increases: moderate stiffness ↑, permeability ↓ modestly, responsiveness to pH ↑; (ii) Pentitol fraction increases: stiffness ↑↑, permeability ↓↓, domain formation likelihood ↑, surface film robustness ↑, and (iii) Hexitol fraction increases: stiffness ↑↑↑, permeability ↓↓↓, strong tendency toward rigid/gel-like surfaces unless screened, strongest surface adsorption/film persistence.

#### 6.4.3. What This Comparison Clarifies for Evolutionary Pathway

Model’s distribution (tetritols abundant, pentitols smaller but significant, hexitols minor) actually makes functional sense: (i) Tetritols supply the “bulk” membrane-forming amphiphiles that are stable but still dynamic; (ii) Pentitols act as a minority “strengthening and functionalizing” component, improving robustness and selectivity, and (iii) Hexitols act as rare but powerful “architectural modifiers” that can nucleate exceptionally stable films or reinforce membranes in harsh niches (alkaline, borate-rich, mineral-rich settings). That is a plausible abiotic version of what biology later did with specialized lipids: most lipids provide the basic bilayer, while minority lipids tune mechanics and function.

## 7. Best Borate-Bridged Amphiphile–Carbohydrate Conjugates for Abiotic Membranes

The topic of “protolipids” and abiotic membranes, especially in the last 3–5 years, has been of great interest to scientists of various specialties, including astrobiologists, chemists, physicists, and others. This growing interest reflects a broader shift toward interdisciplinary approaches in understanding the origin of life [97,98,99,100]. Advances in experimental techniques and theoretical modeling have enabled more detailed exploration of prebiotic self-assembly processes. In particular, studies of non-canonical amphiphiles and environmentally responsive systems have expanded the range of plausible membrane-forming chemistries. At the same time, insights from planetary science and geochemistry have provided increasingly realistic constraints on the environmental conditions under which such systems could emerge [101,102,103,104,105,106,107,108,109]. Together, these developments have transformed the study of abiotic membranes from a largely speculative field into a more experimentally grounded and quantitatively testable area of research.

From an abiotic-membrane perspective, the “best” conjugate is not the most chemically sophisticated, but the one that maximizes key traits under early-Earth conditions: (i) stable self-assembly in saline water, (ii) tolerance to pH and temperature shifts, (iii) mechanical integrity against dilution and shear, and (iv) sufficient functionality (borate–diol binding and sugars) to form selective, hydrated interfaces without destabilization. Comparing batyl/selachyl/chimyl-derived single-tail systems with multi-tail polyol scaffolds (tetritol/pentitol/hexitol) reflects two distinct strategies: interfacial films and micelles versus bilayer membranes.

Single-tail batyl, selachyl, and chimyl conjugates are strong candidates for rapid interfacial organization, as they readily form micelles and monolayers. Their borate–ribose headgroups favor dense surface films at air–water interfaces, mineral surfaces, and droplets—an advantage in heterogeneous prebiotic settings. However, their geometry limits them to high-curvature, leaky assemblies rather than robust bilayers. Selachyl’s unsaturation enhances fluidity, while batyl and chimyl pack more tightly. Overall, these systems are effective proto-boundaries but unlikely to form durable vesicles without mixing with other amphiphiles [1,2].

Tetritol-based conjugates introduce a critical shift: two hydrophobic tails enable stable bilayer formation. Butane-1,2,3,4-tetraol scaffolds support vesicle-capable amphiphiles while retaining borate-binding diols. Their borate–sugar headgroups provide tunability—expanding under alkaline conditions—while salt screening stabilizes bilayers in realistic environments. Tetritol diethers therefore occupy a practical “sweet spot” of structural simplicity and environmental adaptability [1].

Pentitol-based systems further enhance robustness. The additional hydroxyl group increases borate-binding redundancy and headgroup versatility. Two-tail pentitol conjugates resemble tetritols but better maintain functionality across fluctuations. Three-tail variants act as strong membrane stabilizers, increasing cohesion, reducing permeability, and supporting persistent lamellae. While they risk phase complexity under extreme conditions, pentitols—especially 2–3 tail systems—offer superior durability and versatility as abiotic membrane materials.

Hexitol-based conjugates extend this trend but introduce overfunctionality risks. With multiple diol sites and higher tail capacity, they can strongly stabilize membranes but may also promote excessive crosslinking or phase separation. Two-tail hexitols remain viable bilayer formers; three-tail variants excel as stabilizers in harsh conditions; four-tail systems likely function best as reinforcing components rather than primary membrane formers.

Overall, no single system dominates across all conditions. For vesicle formation, 2-tail tetritol and pentitol conjugates are optimal, with pentitols offering greater robustness. For extreme environments, 3-tail pentitol and hexitol systems provide superior stability, with pentitols representing a safer balance and hexitols acting as powerful reinforcers. For early-stage surface organization, single-tail alkylglycerol conjugates remain highly effective, likely serving as initial boundary formers prior to the emergence of stable bilayer membranes.

So, as a final chord in evolutionary-chemistry framing: single-tail alkylglycerols (batyl/selachyl/chimyl) are the best “first boundaries” and interfacial organizers; tetritol diethers are the best first true bilayer lipids; pentitol (2–3 tail) conjugates are the best general-purpose abiotic membrane materials; and hexitol (2–3 tail) conjugates are the best minority reinforcers that can stiffen and seal membranes in extreme niches. If we forced a single “best physicochemical package” across the widest plausible early-Earth environments, I would choose a pentitol-based conjugate with two tails as the dominant bilayer-former, supplemented by a small fraction of three-tail pentitol or hexitol conjugates for mechanical reinforcement, with batyl/selachyl/chimyl conjugates serving as surface-active modulators and film formers. That mixed-composition answer is chemically realistic (prebiotic synthesis produces mixtures), physically optimal (minority components tune mechanics), and evolutionarily plausible (selection acts on stability and persistence of assemblies, not on purity).

## 8. Discussion of the Model of a Borate-Bridged Protolipid Membrane

**Figure 8:**
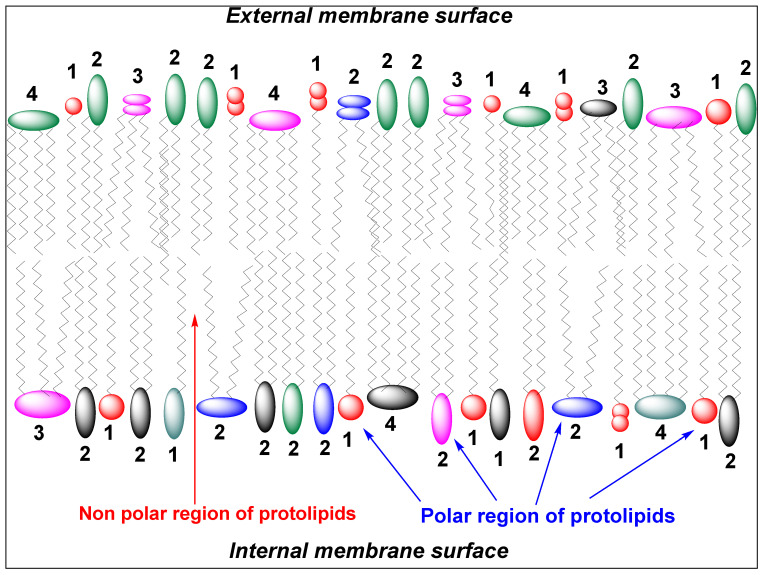
The polar head groups are represented by different colors, reflecting the diversity of carbohydrates that may participate in borate coordination. These sugars include ribose and other pentoses or hexoses capable of forming stable borate esters through their 1,2- or 1,3-diol configurations. The presence of different sugar moieties highlights the potential chemical heterogeneity of early amphiphilic assemblies, which may have incorporated a mixture of polyols and carbohydrates available in prebiotic environments. Such heterogeneity could have contributed to variations in surface charge, hydration properties, and molecular recognition at membrane interfaces.

The schematic representation illustrates (see Figure 8) a conceptual model of an abiotic bilayer membrane constructed from borate-bridged amphiphile–carbohydrate conjugates, which we designate as *protolipids*. In this model, amphiphilic molecules are formed through reversible coordination between borate species and carbohydrate or polyol groups containing vicinal diols. Such borate–diol interactions generate stable yet dynamic linkages that connect hydrophilic sugar head groups to amphiphilic frameworks, allowing the formation of membrane-like assemblies under plausible prebiotic conditions [20].

A central feature of the model is the borate-mediated linkage between carbohydrates and amphiphilic building blocks, replacing the permanent ester or ether bonds of modern phospholipids. Borate ions readily form cyclic esters with diol-containing molecules such as ribose, glycerol, and related sugars. This reversible chemistry enables borate-bridged conjugates with both hydrophilic and hydrophobic domains. Hydrophobic tails drive aggregation via van der Waals interactions, while carbohydrate–borate headgroups interact with water, collectively promoting bilayer self-assembly.

Structural variability arises from the number of hydrophobic tails per molecule (1–4), spanning single-chain surfactant-like amphiphiles to multi-tailed structures. Single-tail molecules tend to form micelles or loosely organized membranes, whereas two-tail protolipids favor bilayers due to their cylindrical geometry. Amphiphiles with three or four tails increase hydrophobic packing and rigidity, stabilizing thicker or more ordered membrane domains.

The coexistence of different tail architectures implies a dynamic, adaptive membrane. Single-tailed species enhance fluidity and curvature, while multi-tailed molecules act as stabilizing anchors. This compositional diversity would allow membranes to respond to changing temperature, pH, and ionic conditions [20].

Borate coordination is central to this behavior, bridging carbohydrate headgroups and enabling supramolecular organization. Because borate–diol interactions are reversible, membranes remain dynamic and capable of continuous reorganization—an advantage in prebiotic environments with ongoing chemical turnover.

Finally, borate introduces a mechanism for molecular selection. Its preference for specific diol geometries, such as those in ribose, could bias incorporation toward certain sugars. This selective stabilization may have enriched particular carbohydrates within membranes, linking compartment formation to broader prebiotic chemical selection processes.

The proposed membrane architecture also suggests a mechanism by which carbohydrate-rich interfaces could have participated in early chemical evolution. Sugar-containing head groups may have facilitated hydrogen bonding networks and interactions with other prebiotic molecules, including nucleobases, peptides, or metal ions. These interactions might have created localized environments conducive to chemical reactions, molecular concentration, and the emergence of primitive metabolic or informational systems [20].

Overall, the figure represents a conceptual framework for an abiotic membrane composed of borate-bridged protolipids, highlighting the structural diversity and dynamic nature of such assemblies. The combination of amphiphilic self-assembly and reversible borate coordination provides a plausible pathway for the formation of primitive membrane structures prior to the emergence of modern phospholipid-based membranes. By integrating polyol-derived head groups with hydrophobic chains of varying number and length, these protolipid systems may have served as transitional architectures linking simple amphiphilic molecules to the complex biomembranes observed in contemporary living cells.

## 9. Conclusions and Future Perspectives

The search for plausible early abiotic membranes requires integrating geochemistry, coordination chemistry, and membrane biophysics. Here, borate-bridged amphiphile–carbohydrate conjugates are presented as coherent candidates for primitive boundary structures. Formed from low-molecular-weight polyols, long-chain alkyl ethers, and borate under alkaline conditions, these systems enable reversible coupling to sugars such as ribose. The resulting architectures combine hydrolytically stable ether-linked hydrophobic domains with multivalent, hydrated, and pH-responsive headgroups, distinguishing them from simple fatty acid vesicles and supporting their role as intermediates toward biological membranes.

Comparative analysis reveals a functional gradient. Single-tail alkylglycerols (batyl, selachyl, chimyl) form micelles and interfacial films, making them effective early surface-bound boundaries. Tetritol- and pentitol-based diethers introduce bilayer formation and broader stability across pH and salinity. Tri-tail pentitol and hexitol derivatives enhance cohesion and mechanical strength, acting as stabilizers in mixed systems. Hexitols offer strong stabilization but risk over-crosslinking and phase separation. Overall, the most favorable scenario involves heterogeneous mixtures dominated by two-tail polyol diethers, supplemented by tri-tail stabilizers and surface-active alkylglycerols, providing resilience, permeability control, and adaptability.

Beyond structure, these systems link membrane assembly with carbohydrate stabilization. Borate selectively binds vicinal diols, favoring certain sugar conformations and enabling pre-enzymatic concentration at interfaces. This coupling suggests membranes may have acted as chemically active sites influencing molecular selection, not just passive barriers.

A key implication is that early membranes operated within “stability windows” rather than universal rules. Because borate interactions depend on pH, ionic strength, and water activity, these systems likely exhibited transitions between films, micelles, and bilayers across specific conditions. This leads to testable predictions: (i) sugar association should track borate speciation and alter surface charge and curvature; (ii) increased ionic strength should stabilize bilayers by screening repulsion; and (iii) small fractions of tri-tail components should enhance mechanical strength up to a limit before phase separation occurs.

Future work should experimentally validate these behaviors through phase mapping, aggregation measurements, cryo-EM imaging, mechanical testing, and borate complexation studies (e.g., ^11^B NMR). Mixed systems and mineral-supported assemblies are particularly important targets for testing environmental realism.

More broadly, this framework suggests membrane evolution may have involved intermediate systems based on ether-linked polyols and reversible coordination chemistry, rather than a direct transition from fatty acids to phospholipids. Such systems could provide both durability and chemical selectivity prior to biological lipid synthesis.

In conclusion, borate-bridged amphiphile–carbohydrate conjugates represent a plausible and experimentally testable model for abiotic membranes, expanding our understanding of how early chemical systems could organize into stable, functional boundaries.

## Figures and Tables

**Table 1:** Summary of borate-bridged amphiphile systems and their physicochemical properties.

System/Amphiphile Type	Molecular Composition	Approx. Molecular Weight	Packing Parameter (Inferred)	Headgroup Characteristics	Expected Assembly Behavior
Batyl alcohol (1)	C18 ether glycerol (1 alkyl chain, 2 OH)	~330–350 g/mol	<1/3 (cone-shaped)	Small, neutral diol	Micelles, interfacial films
Selachyl alcohol (2)	C18:1 ether glycerol (unsaturated chain)	~330–350 g/mol	<1/3 (cone-shaped, more fluid)	Small, neutral diol	Micelles, fluid interfacial layers
Chimyl alcohol (3)	C16 ether glycerol	~300–320 g/mol	<1/3 (cone-shaped)	Small, neutral diol	Micelles, monolayers
Borate–glycerol complex	Ether glycerol + borate (cyclic ester)	+~40–60 g/mol	↓ packing (larger headgroup)	Expanded, anionic/neutral (pH-dependent)	Micelles, stabilized interfaces
Borate–amphiphile–sugar conjugate	Ether glycerol + borate + carbohydrate (e.g., ribose)	~450–600 g/mol (variable)	<1/3 (strongly cone-shaped)	Large, hydrated, H-bonding, charged (pH-dependent)	Stable micelles, dense interfacial films
Mixed systems (with fatty acids or diacyl lipids)	Mixed amphiphile compositions	Variable	Tunable (0.3–1)	Heterogeneous	Membrane modulation, curvature control, mixed aggregates

**Table 2:** Qualitative molecular-orbital data that would apply to a typical borate-bridged amphiphile–carbohydrate conjugate.

System	Key Functional Groups	Dominant Orbitals (HOMO/LUMO)	Boron Interaction	Conjugation/Delocalization	Polarity & Electronic Effects	Structural Implication
Batyl alcohol	Ether (–O–), primary OH	HOMO: O lone pairs (n_O); LUMO: σ* (C–O)	None	Localized; no extended conjugation	Moderately polar head, nonpolar tail	Typical amphiphile; favors micelle formation
Selachyl alcohol	Ether, OH, C=C	HOMO: π (C=C) + n_O; LUMO: π* (C=C)	None	Weak π-delocalization at alkene	Slightly more polarizable due to π-system	Increased fluidity in aggregates
Chimyl alcohol	Ether, primary OH	HOMO: n_O; LUMO: σ* (C–O)	None	Fully localized	Similar to batyl but shorter chain	Slightly less hydrophobic packing
Borate–polyol (butane-1,2,3,4-tetraol)	Multiple OH, B(OH)_3-derived	HOMO: O lone pairs; LUMO: empty p orbital on B	Strong Lewis acid–base (B ← O)	Delocalization across B–O network (σ + partial π donation)	Highly polar, electron-deficient boron center	Forms cyclic borate esters
Batyl–borate conjugate	Ether + borate ester + polyol	HOMO: delocalized n_O over B–O; LUMO: B-centered p orbital	O→B donation stabilizes complex	Moderate delocalization over B–O–C network	Increased polarity at headgroup	Promotes bilayer over micelle
Selachyl–borate conjugate	Ether + C=C + borate ester	HOMO: π (C=C) + n_O (B–O); LUMO: π* + B p orbital	Dual interaction (π-system + B center)	Enhanced delocalization (π + B–O)	More polarizable, responsive electronic structure	Flexible, dynamic membrane domains
Chimyl–borate conjugate	Ether + borate ester	HOMO: n_O (delocalized over B–O); LUMO: B p orbital	Strong O→B coordination	Similar to batyl but slightly reduced due to chain length	Polar head, less hydrophobic stabilization	Slightly less stable bilayer packing

**Table 3:** Qualitative Molecular-Orbital Characteristics of the Borate-Bridged Amphiphile–Carbohydrate Conjugate.

Molecular Region	HOMO Contribution	LUMO Contribution	Electronic/Structural Role
Non-coordinated carbohydrate oxygen atoms	High	Low–moderate	Primary electron-donor sites
Borate-coordinated oxygen atoms	Moderate	Moderate	Electron density reduced due to B–O bonding
Boron center	Low	High	Electron-accepting and polarizing center
Adjacent carbohydrate carbons	Moderate	Moderate	Part of electronically active headgroup
Long amphiphilic alkyl chain	Very low	Very low	Hydrophobic anchor with minimal orbital involvement

**Table 4:** Compact property table: tri-C18 pentitol borate–ribose conjugate *.

Property	Best Estimate/Expected Range	Key Dependencies (pH, Salt)	What You Should See Experimentally
Molecular weight (MW)	~1.05–1.15 kDa for “tri-C18 pentitol + borate + 1 ribose” (exact value depends on borate hydration/protonation and whether the boron center is anionic with counterion association)	Higher pH favors anionic borate forms; counterion association (Na^+^, K^+^, etc.) can increase apparent mass in MS/complexes	MS (if available) shows a cluster of closely spaced species (±H/±OH/±counterion); ^11^B NMR confirms boron speciation rather than mass
Solubility/aggregation threshold	Very low monomer solubility; extremely low CAC/CMC (aggregation at very low μM or below is plausible) because of three C18 chains	Salt may *either* stabilize dispersed vesicles by screening headgroup repulsion *or* promote phase separation if it dehydrates the headgroup	DLS shows particles already present at very low concentration; concentration dependence is weak once above CAC
Dominant morphology: pH ~6–8	Condensed lamellae/multilamellar aggregates; possible precipitation or waxy phases if headgroup is not sufficiently hydrated	Weak borate bridging → smaller headgroup → stronger hydrophobe dominance → lower curvature, tighter packing	DLS: larger, polydisperse aggregates; cryo-TEM: stacked lamellae or dense sheets; zeta potential closer to neutral
Dominant morphology: pH ~8.5–10.5	Lamellar bilayers and multilamellar vesicles are most likely; vesicle-like objects more stable because borate–diol binding promotes a larger hydrated headgroup	Borate bridging favored; ionic strength tunes vesicle size (screening increases packing and stability)	DLS: stable size distributions; cryo-TEM: multilamellar vesicles/lamellae; ^11^B NMR shows dominant tetrahedral borate complex signals consistent with diol binding
Dominant morphology: pH ≥ 10.5 (low salt)	More “expanded” headgroups → smaller vesicles/higher curvature or coexistence with non-lamellar phases at higher concentration	Strong borate + high hydration/charge → repulsion increases curvature; low salt fails to screen	DLS: smaller mean size and/or bimodal distributions; zeta potential more negative; cryo-TEM: smaller vesicles and occasional non-lamellar textures
Dominant morphology: high salt (any pH, esp. alkaline)	Tighter lamellae, thicker/less permeable assemblies, potentially more multilamellar stacking; at very high salt, risk of dehydration-driven aggregation	Screening lowers electrostatic repulsion but can also reduce headgroup hydration at extremes	DLS: larger aggregates/more stacking; cryo-TEM: increased lamellar order; Langmuir films become more condensed
Headgroup charge (zeta potential)	Near neutral to mildly negative at pH 6–8; more negative at alkaline pH, where borate complexes are favored	Strongly pH-dependent; salt reduces magnitude by screening	Zeta potential vs. pH curve: becomes more negative as pH increases; magnitude drops at higher ionic strength
Predicted mechanics	High bending modulus (stiff), low permeability, strong film persistence due to tri-tail cohesion; stiffness increases with stronger headgroup networking (borate binding) and with salt-screened packing	pH increases headgroup size/charge (can soften packing if unscreened); salt generally increases mechanical robustness by reducing repulsion	Cryo-TEM shows thick/rigid lamellae; Langmuir isotherms show high collapse pressure and condensed phases
Interfacial film behavior	Very strong monolayer formation at air–water and strong adsorption to hydrophilic solids (silica/clay)	Alkaline pH (with borate) increases headgroup hydration; salt shifts compressibility	Langmuir π–A isotherms: early lift-off, condensed state at moderate compression, high collapse pressure; hysteresis indicates robust films
Boron binding/pH titration signature	Boron transitions from weakly bound/boric-acid-like at low pH to tetrahedral borate diol complexes at alkaline pH	Controlled by boron pKa (shifted by ionic strength) and diol availability/competition	^11^B NMR pH titration: shift from trigonal boric acid signal to tetrahedral borate/diol-complex signals; stronger complex signature near pH ~9–11

* (Borate-bridged amphiphile–carbohydrate conjugate with a pentane-1,2,3,4,5-pentaol backbone and three C18 ether tails).

## Data Availability

No new data were created or analyzed in this study. Data sharing is not applicable to this article.
